# Toward high-current-density and high-frequency graphene resonant tunneling transistors

**DOI:** 10.1038/s41467-025-58720-7

**Published:** 2025-05-23

**Authors:** Zihao Zhang, Baoqing Zhang, Yifei Zhang, Yiming Wang, Patrick Hays, Seth Ariel Tongay, Mingyang Wang, Hecheng Han, Hu Li, Jiawei Zhang, Aimin Song

**Affiliations:** 1 Institute of Nanoscience and Applications, Southern University of Science and Technology, Shenzhen, China; 2 Shandong Technology Center of Nanodevices and Integration, School of Integrated Circuits, Shandong University, Jinan, China; 3 School for Engineering of Matter, Transport and Energy, Arizona State University, Tempe, AZ USA; 4 Department of Electrical and Electronic Engineering, University of Manchester, Manchester, UK

**Keywords:** Electronic devices, Electronic properties and devices

## Abstract

Negative differential resistance (NDR), a peculiar electrical property in which current decreases with increasing voltage, is highly desirable for multivalued logic gates, memory devices, and oscillators. Recently, 2D quantum-tunneling NDR devices have attracted considerable attention because of the inherent atomically flat and dangling-bond-free surfaces of 2D materials. However, the low current density of 2D NDR devices limits their operating frequency to less than 2 MHz. In this study, graphene/hexagonal boron nitride (h-BN)/graphene resonant tunneling transistors (RTTs) were fabricated using graphene and h-BN barriers with different numbers of atomic layers, showing a mechanism enabling the observation of NDR in high current density devices. A triangular etching approach was proposed to suppress the effects of graphene–metal contact resistance and graphene sheet resistance, enabling pronounced NDR effect even in a 2D tunneling device with a single atomic layer h-BN barrier. A room-temperature peak current density up to 2700 μA/μm^2^ and operational frequencies up to 11 GHz were achieved, demonstrating the potential of 2D quantum NDR devices for applications in high-speed electronics.

## Abstract (web summary)

2D devices with negative differential resistance are desired for multivalued logic applications, but they are normally limited by low current densities and operational frequency. Here, the authors report graphene/hexagonal boron nitride/graphene resonant tunnelling transistors with room temperature peak current density up to 2700 μA/μm^2^ and operational frequencies up to 11 GHz.

## Introduction

Two-dimensional materials can be vertically stacked in van der Waals heterostructures to realize versatile applications such as superconductive electronics^1–3^, ferroelectronics^4–6^, optoelectronics^7–9^, and spintronics^10^. Negative differential resistance (NDR), a peculiar electrical property in which the current decreases with increasing voltage, is a key element in high-frequency electronics and is highly desirable in 2D devices. This property can be realized using quantum resonant tunneling diodes (RTDs), which rely on resonant tunneling through a quantized state between (usually two) barriers, as well as Esaki tunnel diodes, which are based on band-to-band tunneling at the interface between two materials or the same material but with opposite dopings. Two-dimensional materials are highly suitable for building tunneling devices owing to their easily formed heterostructures and dangling-bond-free interfaces. Meanwhile, 2D material resonant tunneling devices can be controlled by a gate and are therefore also known as resonant tunneling transistors (RTTs)^11,12^. Since the first realization in a graphene/hexagonal boron nitride (h-BN)/graphene heterostructure in 2013 (ref. ^11^), 2D quantum-tunneling NDR devices have attracted considerable attention. NDR has been observed in RTDs (or RTTs)^12–25^ and Esaki tunnel diodes (or tunnel transistors)^26–40^ with various 2D materials and their combinations, and its potential for use in multivalued logic gates^28,33,38,39^, memory devices^15,36^, and oscillators^12^ has been demonstrated. However, the reported operating frequency of 2D NDR devices, which is a key metric in both logic and analog electronics, has so far been limited to 2 MHz (ref. ^12^). This is attributed to their low current densities, which correspond to high resistances and thereby long RC times and low operating speeds. Thus far, the highest reported peak current density of 2D quantum-tunneling NDR devices is 10 μA/μm^2^ (ref. ^22^). Thus, the limited current density of 2D NDR devices limits their applications, and overcoming this limitation is a major research challenge.

Conventional III–V semiconductor RTDs, which are based on double-barrier resonant tunneling, have been demonstrated as terahertz (up to 1.98 THz) oscillators using their NDR characteristics^41–45^. Two-dimensional RTTs based on single-barrier resonant tunneling are expected to have much higher intrinsic device speeds owing to the absence of the carrier dwell time in the central quantum well^11^. However, the operating frequency of 2D RTTs is so far limited owing to their low current densities, whereas the peak current densities of III–V RTD terahertz oscillators are typically higher than 10^3^ μA/μm^2^. This is somewhat unexpected because graphene has a zero bandgap, the highest carrier mobility, and a very low sheet resistance among 2D materials, thus rendering it useful as a tunneling electrode for high current densities and high frequencies. It is therefore timely to study the limiting mechanism of 2D RTTs and thereby develop a method for improving the current density of graphene/h-BN/graphene RTTs.

In this study, the effects of different numbers of graphene and h-BN barrier atomic layers and different geometries on the tunneling characteristics were investigated. After achieving a high tunneling current density with a thin barrier, the NDR effect was found to vanish. Further studies revealed that the tunneling resistance was much smaller than the parasitic resistance. The finding enabled us to design a triangular etching approach to suppress the effect of the parasitic resistance and revive the NDR characteristics. A series and transmission-line resistance model was developed to provide an analytical expression for the effective transmission length of the tunneling current. With the understanding and the modeling, the resonant-tunneling-based NDR phenomenon was revived in graphene RTTs with as few as a single layer of h-BN barrier. Furthermore, the monolayer h-BN barrier device achieved a room-temperature peak current density of 2700 μA/μm^2^, which is already comparable to the peak current densities of III–V RTDs. The high-frequency performance of the graphene RTT was demonstrated at frequencies up to 11 GHz.

## Results and discussion

### Effect of the number of h-BN barrier layers

**Fig. 1:**
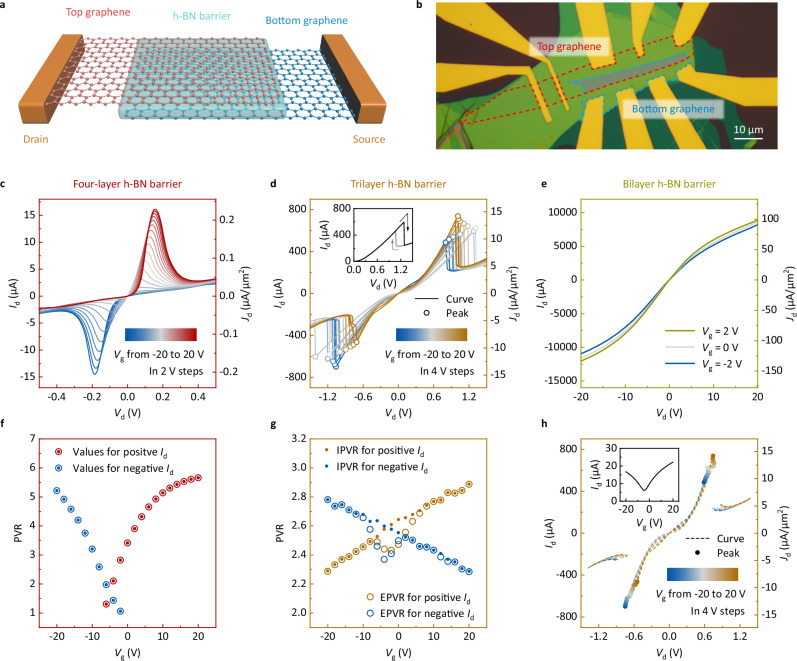
Output characteristics and peak-to-valley ratio (PVR) of monolayer graphene resonant tunneling transistors (RTTs) with different numbers of hexagonal boron nitride (h-BN) barrier atomic layers. **a** Structure schematic of graphene RTT. **b** Optical micrograph of a fabricated graphene RTT. The red and blue dashed lines indicate the ranges of the top and bottom graphene flakes, respectively. **c**–**g** Output characteristics (**c**–**e**) and PVRs (**f**, **g**) of a four-layer (**c**, **f**), a trilayer (**d**, **g**), and a bilayer (**e**) h-BN barrier RTT, in which the drain current *I*_d_ and drain current density *J*_d_ change with the drain voltage *V*_d_ under different gate voltages *V*_g_. The inset of (**d**) shows the output characteristics under *V*_g_ = −4 V. The arrows indicate forward and backward sweeps. **h** Output characteristics of the trilayer h-BN barrier RTT with the series resistance deducted. The inset shows the transfer characteristics of a graphene field-effect transistor (FET) under *V*_d_ = 10 mV, which is used to deduct the series resistance.

To improve the RTT current density, the effect of the number of atomic layers of the h-BN tunneling barrier was evaluated. A structural schematic of the graphene RTT is shown in Fig. 1a. The top and bottom graphene flakes were aligned in a crystallographic orientation and separated by several atomic layers of h-BN barrier. An optical micrograph of a fabricated RTT is shown in Fig. 1b. A heavily p-doped Si/SiO_2_ wafer was used as the substrate. Resonant tunneling requires momentum and energy matching^12,22,25^. Here, momentum matching was ensured by aligning the graphene crystallographic orientations, and energy matching was achieved by adjusting the drain and gate biases. Monolayer graphene RTTs with four-layer, trilayer, and bilayer h-BN barriers were fabricated. The output characteristics of the three devices are shown in Fig. 1c–e. In Fig. 1c, the four-layer h-BN barrier device exhibited typical NDR behavior, where the drain current *I*_d_ decreased with the drain voltage *V*_d_ in a certain regime. The gate voltage *V*_g_ modulated the amplitude and position of the current peak. Additionally, *V*_d_ of the current peak increased approximately linearly with *V*_g_. The electrical characteristics of the four-layer h-BN barrier device can be explained by the standard graphene RTT operating principle^11,12^ and are consistent with previously reported experimental results^11,12,23,25^. As shown in Fig. 1d, the trilayer h-BN barrier device exhibited different behaviors. Firstly, the output curves dropped or increased abruptly in the NDR region and exhibited hysteresis between the forward and backward sweeps, as shown in the inset of Fig. 1d. This phenomenon has been reported in previous studies and is attributed to the series resistance^18^. Secondly, *V*_d_ at the peak did not vary monotonically with *V*_g_ when *V*_g_ was near 0 V (the current peaks are highlighted by hollow circles in Fig. 1d). It is also noted that the trilayer h-BN barrier RTT exhibited an increased peak current density (peak current divided by active area, which refers to the area where two graphene flakes overlap) of 15 μA/μm^2^. However, when the number of h-BN layers was further reduced, as shown in Fig. 1e, the bilayer h-BN barrier device did not exhibit any NDR characteristics.

Peak-to-valley ratio (PVR) has been used in previous studies as a key metric for evaluating NDR performance. In Fig. 1f, g, the PVRs of the four-layer and trilayer RTTs are plotted. In this study, two types of PVR were introduced to evaluate RTT performance. One is called external PVR (EPVR), which is defined as the ratio of the peak current to the valley current of the forward sweeping curve, and refers to the PVR measured between two external metal electrodes in a single (forward) sweep. The other is called internal PVR (IPVR), which is defined as the ratio of the forward-sweeping peak current to the backward-sweeping valley current, and refers to the PVR of only the graphene-overlapping region (where tunneling occurs) in the device that is not affected by the series resistance. The EPVR is always lower than or equal to the IPVR. For the four-layer h-BN barrier device, as shown in Fig. 1f, the output curve was almost unaffected by the series resistance, and thus, EPVR was equal to IPVR. The trilayer device had a more pronounced difference between the EPVR and IPVR when *V*_g_ was near −4 V, as shown in Fig. 1g. This result suggests that graphene was close to its Dirac point at *V*_g_ = −4 V, thus increasing the series resistance. The inset of Fig. 1h shows the transfer curve measured from the graphene used in the trilayer h-BN barrier device. The internal output characteristics of the trilayer RTT, without the effect of the series resistance estimated by the transfer curve, are plotted in Fig. 1h (the current peaks are highlighted by solid circles). These curves exhibit a trend similar to the characteristics of the four-layer device (Fig. 1c), indicating that the effect of the series resistance is noteworthy for RTTs with thinner barrier layers.

Two additional trilayer h-BN barrier devices with different crystallographic orientation twist angles of the two graphene flakes are shown in Supplementary Fig. 1. Under the influence of the series resistance, the distribution of current peaks at different *V*_g_ values was “I”-shaped, “J”-shaped, or “>”-shaped, corresponding to a small, medium, or large twist angle, and the transfer characteristics of a graphene FET can deduct the series resistance for all the devices. The calculated results, shown in Supplementary Fig. 2, agree closely with the experimental results. At a larger twist angle of approximately 6°, as shown in Supplementary Fig. 3, the device exhibited a weak gate modulation, whereas the peak current density was not found to be significantly correlated with the twist angle. Additionally, a four-probe measurement^18^ can provide a more direct demonstration of the series resistance reduction than the use of a graphene FET, as shown in Supplementary Fig. 4. However, it can deduct only the portion of the graphene–metal contact resistance in the series resistance rather than all of it.

### Etching process

**Fig. 2:**
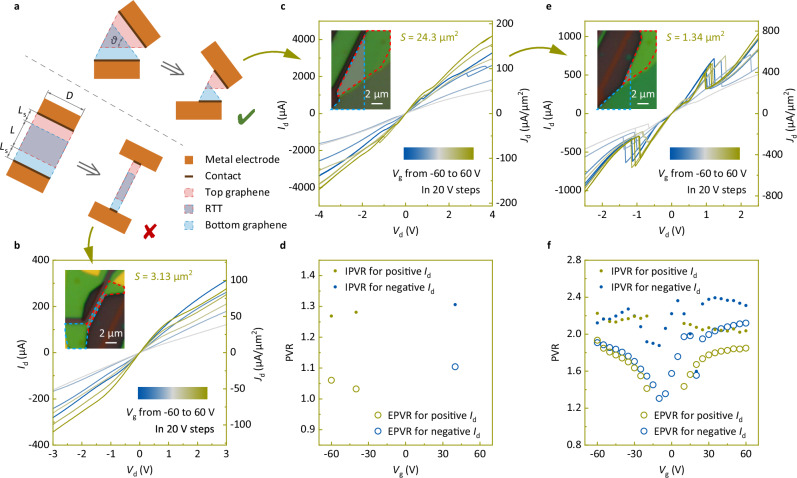
Negative differential resistance (NDR) characteristics revived with etching. **a** Schematics of two etching approaches, where *θ* is the unetched corner of the graphene-overlapping region, *L* is the length of the graphene-overlapping region, and *L*_s_ is the graphene length outside the overlapping region. **b** Output characteristics of the bilayer h-BN barrier RTT after etching using the rectangular etching approach, in which the drain current *I*_d_ and drain current density *J*_d_ change with the drain voltage *V*_d_ under different gate voltages *V*_g_. **c**–**f** Output characteristics (**c**, **e**) and PVR (**d**, **f**) of the device after the first (**c**, **d**) and second (**e**, **f**) etching steps using the triangular etching approach. The PVR includes two types: the external PVR (EPVR) and the internal PVR (IPVR). The insets of (**b**, **c**, **e**) show optical micrographs of the device after the corresponding etching step. The red and blue dashed lines indicate the ranges of the top and bottom graphene flakes, respectively.

To reveal the NDR characteristics in the bilayer h-BN barrier RTT, the effect of the series resistance must be reduced. Therefore, in this study, an inductively coupled plasma (ICP) etching process was introduced. A schematic is shown in Fig. 2a, in which two etching approaches are illustrated: one is etching from two sides to make the graphene-overlapping region a narrower rectangle (see the lower left of Fig. 2a), whereas the other is etching from one side to create a smaller triangle (see the upper right of Fig. 2a). Two etching approaches were used on the same device to minimize the errors arising from device consistency. The device without NDR characteristics shown in Fig. 1e had an area of *S* = 90.2 μm^2^ in the graphene-overlapping region. Subsequently, it was cut into two parts by ICP etching. The active area of the rectangular part was etched to 3.13 μm^2^. The output characteristics are shown in Fig. 2b, and still no NDR was observed. The triangular part was initially etched to 24.3 μm^2^, and the output characteristics and PVR are shown in Fig. 2c, d. NDR characteristics were observed at several different *V*_g_ values, although the highest PVR was only 1.3. The output characteristics and PVR after further etching the device to 1.34 μm^2^ are shown in Fig. 2e, f. The device exhibited clear NDR regions, and the PVR increased to 2.4. Three additional etching processes are shown in Supplementary Figs. 5–7. These results indicate that the absence of NDR for the bilayer h-BN barrier device shown in Fig. 1e was not due to the poor resonant tunneling performance but rather the much lower tunneling resistance compared with that of the trilayer device, and the triangular etching approach could suppress the effect of the series resistance more effectively than the rectangular etching approach. However, the peak current density and IPVR did not change with the series resistance, as observed by comparing Fig. 1d, h. This result is different from the phenomenon observed in Fig. 2c–f, where the peak current density and IPVR increased with etching. Therefore, during the etching process, factors other than the series resistance should also play a critical role.

### Series and transmission-line resistance calculations

**Fig. 3:**
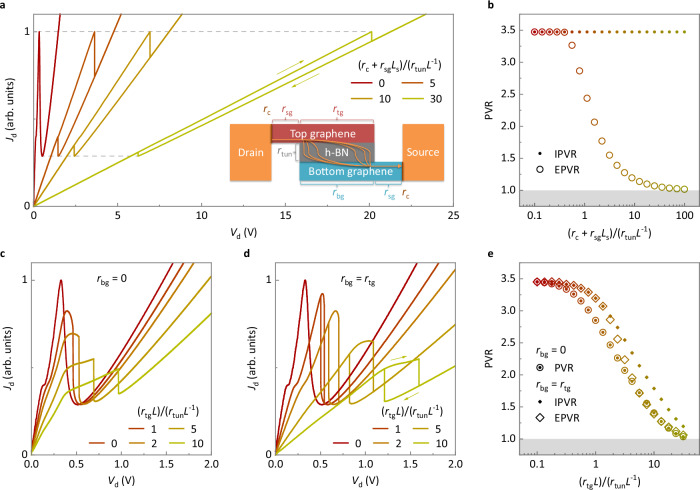
Numerical calculations of the effects of the series and transmission-line resistances on NDR performance. **a** Output characteristics of RTT in which the drain current density *J*_d_ changes with the drain voltage *V*_d_ with different series-to-tunneling resistance ratios. The inset shows a cross-sectional schematic of the RTT with the resistances and current distribution marked, where *r*_tun_ is the tunneling resistance per unit area, *r*_c_ is the contact resistance per unit width between the metal and the graphene, *r*_sg_ is the graphene sheet resistance outside the overlapping region, *r*_tg_ is the top graphene sheet resistance in the overlapping region, *r*_bg_ is the bottom graphene sheet resistance in the overlapping region. **b** PVR as a function of the series-to-tunneling resistance ratio. **c**, **d** Output characteristics with different transmission-line-to-tunneling resistance ratios. **e** PVR as a function of the transmission-line-to-tunneling resistance ratio. The arrows in (**a**, **d**) indicate forward and backward sweeps.

A series and transmission-line resistance model and numerical calculations were introduced to further understand the operating mechanism of the graphene RTT. The dimensions of the RTT are shown in the top-view schematic in Fig. 2a, and the inset of Fig. 3a shows the cross-sectional schematic and different resistances, including the tunneling resistance per unit area *r*_tun_ (in Ω μm^2^), contact resistance per unit width *r*_c_ (in Ω μm) between the metal and the graphene, graphene sheet resistance *r*_sg_ (in Ω/sq) outside the overlapping region, top graphene sheet resistance *r*_tg_ (in Ω/sq) in the overlapping region, and bottom graphene sheet resistance *r*_bg_ (in Ω/sq) in the overlapping region. For comparison, the units were all unified to Ω μm using the length *L* of the graphene-overlapping region and the graphene length *L*_s_ outside the overlapping region, i.e., *r*_tun_*L*^−1^, *r*_c_, *r*_sg_*L*_s_, *r*_tg_*L*, and *r*_bg_*L*. Here, *r*_c_ and *r*_sg_*L*_s_ are in series with the graphene-overlapping region and are collectively referred to as the series resistance. By contrast, *r*_tg_*L* and *r*_bg_*L* are neither in series nor in parallel with *r*_tun_*L*^−1^ but are distributed as in a transmission line and are collectively referred to as the transmission-line resistance. Using the equations described in Supplementary Section 4 and setting both the series and transmission-line resistances to zero, the initial current density–voltage (*J*–*V*) curve of the graphene RTT with a PVR of approximately 3.5 (red curves in Fig. 3a, c, d) was calculated numerically. Subsequently, by considering *V*_d_/*J*_d_ at the current density peak of the *J*–*V* curve as the tunneling resistance and introducing the series resistance into the *J*–*V* curve, the output characteristics and PVR were calculated, as shown in Fig. 3a, b. The introduced series resistance shifted the output curve toward a higher *V*_d_ but did not affect *J*_d_. The EPVR decreased when the series resistance became dominant, whereas the IPVR remained unchanged. These results agree closely with those shown in Fig. 1d, g, h. When the series resistance was much more pronounced than the tunneling resistance, the EPVR approached 1, and NDR occurred only at high drain voltages, causing difficulties in the experimental observations. This phenomenon explains why no NDR was observed in the bilayer h-BN device (Fig. 1e). For the transmission-line resistance, two special cases, *r*_bg_*L* = 0 and *r*_bg_*L* = *r*_tg_*L*, were used to represent the general case, where *r*_tg_*L* and *r*_bg_*L* are two unequal and nonzero values. By introducing a certain transmission-line resistance and setting the series resistance to zero, the output characteristics and PVR were calculated as shown in Fig. 3c–e. In both cases, the curves exhibited similar trends when the transmission-line resistance increased, except that no hysteresis was observed when *r*_bg_*L* = 0. It is noteworthy that both the peak current density and IPVR decreased with the transmission-line resistance, which is different from the effect of the series resistance but consistent with the phenomenon shown in Fig. 2c–f. This result suggests that both the series and transmission-line resistances affected the performance of the bilayer h-BN barrier RTT and that the introduction of the etching process reduced the effects of both the series and transmission-line resistances.

For the transmission-line resistance, further analytical calculations were performed to obtain the expressions for the current and bias distributions, as presented in Supplementary Section 5. The calculated results indicate that the transmission-line resistance caused inhomogeneous distributions of current and bias. The inhomogeneous distribution of the bias resulted in the loss of IPVR, and the inhomogeneous distributions of the current and bias together resulted in the loss of the peak current density, which is indicative of how the transmission-line resistance affects the IPVR and peak current density. The calculated results also indicate that the tunneling current was distributed approximately homogeneously in the h-BN barrier with a small device length *L*. When *L* was large, the tunneling current was concentrated in the range of $\sqrt{{r}_{{{\rm{tun}}}}/\left({r}_{{\mbox{tg}}}+{r}_{{\mbox{bg}}}\right)}$ on each side, close to the source and drain. The effective transmission length of the current can be expressed as

1 $${L}_{{{\rm{eff}}}}=\left\{\begin{array}{cc}L,& \quad L \, < \, 2 \sqrt{r_{{{\rm{tun}}}}/(r_{{{\rm{tg}}}}+r_{{{\rm{bg}}}})}\\ 2 \sqrt{r_{{{\rm{tun}}}}/(r_{{{\rm{tg}}}}+r_{{{\rm{bg}}}})},&\quad L \geq 2 \sqrt{r_{{{\rm{tun}}}}/(r_{{{\rm{tg}}}}+r_{{{\rm{bg}}}})}\end{array}\right.$$

Excellent NDR performance requires homogeneous current distribution. Therefore, the device length should satisfy

2 $$L\, \ll \, 2\sqrt{{r}_{{{\rm{tun}}}}/\left({r}_{{{\rm{tg}}}}+{r}_{{{\rm{bg}}}}\right)}.$$

This rule provides a reference for designing RTT devices.

The mechanism by which the etching process improves device performance can be explained by a set of equations. By introducing the width *D* of the device and unifying the units in Ω, for the rectangular etching approach, we obtain

3 $${{{\rm{Tunneling}}}}\; {{{\rm{resistance}}}}={r}_{{{\rm{tun}}}}{L}^{-1}{D}^{-1}\propto {D}^{-1},$$

4 $${{{\rm{Series}}}}\; {{{\rm{resistance}}}}=({r}_{{{\rm{c}}}}+{r}_{{{\rm{sg}}}}{L}_{{{\rm{s}}}}){D}^{-1}\propto {D}^{-1},$$

5 $${{{\rm{Top}}}}/{{{\rm{bottom}}}}\;{{{\rm{transmission}}}}\;{{{\rm{line}}}}\;{{{\rm{resistance}}}}=r_{{{\rm{tg}}}/{{\rm{bg}}}}LD^{-1}\propto D^{-1}.$$

For the triangular etching approach, given that the average length of the graphene-overlapping region is *L* = *D* sin (*θ*/2) (see Fig. 2a), we obtain

6 $${{{\rm{Tunneling}}}}\; {{{\rm{resistance}}}}={r}_{{{\rm{tun}}}}{D}^{-2}{(\sin (\theta /2))}^{-1}\propto {D}^{-2},$$

7 $${{{\rm{Series}}}}\; {{{\rm{resistance}}}}=({r}_{{{\rm{c}}}}+{r}_{{{\rm{sg}}}}{L}_{{{\rm{s}}}}){D}^{-1}\propto {D}^{-1},$$

8 $${{\rm{Top}}}/{{\rm{bottom}}\; transmission}\;{{\rm{line}}\; resistance}=	 {r}_{{{\rm{tg}}}/{{\rm{bg}}}}\sin (\theta /2),\\ 	{{\rm{independent}}\; of\; }{D}.$$

In the rectangular etching approach, the tunneling, series, and transmission-line resistances increased at the same rate when the width of the device was reduced. In the triangular etching approach, the tunneling resistance increased the fastest among the three resistances when the device width was reduced. Therefore, the triangular etching approach can make the tunneling resistance more pronounced, thus reviving the NDR characteristics of the bilayer h-BN barrier RTT. Equation (2) can be rewritten with the area expression as

9 $${{{\rm{Area}}}}={LD}=\frac{{L}^{2}}{\sin (\theta /2)}\ll \frac{4}{\sin (\theta /2)}\frac{{r}_{{{\rm{tun}}}}}{{r}_{{{\rm{tg}}}}+{r}_{{{\rm{bg}}}}},$$

which is the condition for overcoming the effect of the transmission-line resistance using the rectangular etching approach.

It is noted that the defects in h-BN can significantly affect the electrical characteristics of 2D materials in contact with it^46,47^, and in particular, may lead to *I*–*V* curves with step-like features^48^ or negative differential resistance^49^ in tunneling devices. Therefore, scanning transmission electron microscopy (STEM) imaging on h-BN samples was performed in this study. A total of 20 images were captured, one of which is shown in Supplementary Fig. 9, and no obvious defects were found in any of the images. Moreover, the different devices presented in Figs. 1d, h and 2, Supplementary Fig. 1, and Supplementary Figs. 5–7 agree with the modeling without considering defects presented in Fig. 3 and Supplementary Fig. 2, indicating that the defect density may be relatively low so that any defect-related tunneling in this study was not dominant.

Notably, the transmission-line resistance concept and triangular etching technique proposed in this study are unique to graphene-based resonant tunneling structures. Conventional III–V RTDs adopt entirely vertical configurations, where transmission-line resistance is absent. In contrast, graphene-based RTTs require horizontal introduction of graphene electrodes into the active region prior to vertical tunneling, thereby necessitating the introduction of transmission-line resistance and triangular etching. The horizontal integration of graphene electrodes is specifically designed to accommodate vertical gate modulation, which constitutes the fundamental distinction between RTTs and RTDs.

### Toward high current density

**Fig. 4:**
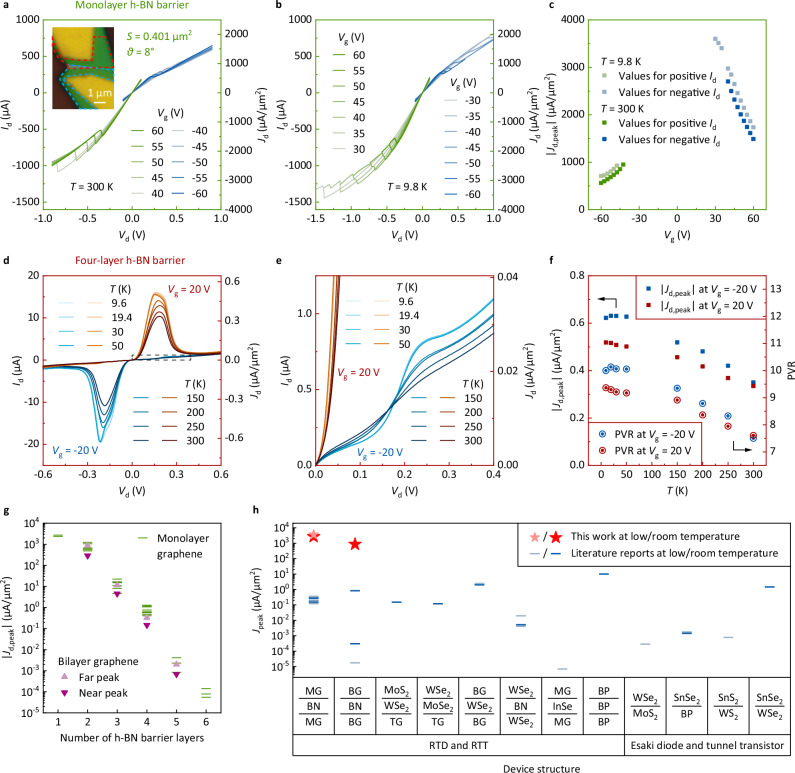
Toward high peak current density. **a**–**c** Output characteristics at 300 K (**a**), output characteristics at 9.8 K (**b**), and peak current density | *J*_d,peak_| (**c**) of a monolayer graphene, monolayer h-BN barrier RTT fabricated with the highest peak current density in this study. Measurements were not performed in *V*_d_ and *V*_g_ regions where NDR signals are not expected. The inset of (**a**) shows an optical micrograph of the device after fabrication and etching. The red and blue dashed lines indicate the ranges of the top and bottom graphene flakes, respectively. **d** Output characteristics of a monolayer graphene, four-layer h-BN barrier RTT at different temperatures *T*. **e** Partial enlargement within the gray dashed box in (**d**). **f** Peak current density and PVR as a function of *T*. **g** Room-temperature peak current density for different numbers of graphene and h-BN barrier atomic layers. **h** Comparison of the highest peak current densities of monolayer and bilayer graphene RTTs achieved in this study with those of 2D quantum-tunneling NDR devices in the literature (where RTD is resonant tunnling diode). References: MG (monolayer graphene)/BN (boron nitride)/MG^11,12,23^, BG (bilayer graphene)/BN/BG^14,17^, TG (trilayer graphene)/WSe_2_/MoS_2_^16^, TG/MoSe_2_/WSe_2_^16^, BG/WSe_2_/BG^18^, WSe_2_/BN/WSe_2_^19,24^, MG/InSe/MG^21^, BP (black phosphorus)/BP/BP (twisted BP homostructure)^22^, MoS_2_/WSe_2_^26^, BP/SnSe_2_^27^, WS_2_/SnS_2_^32^, and WSe_2_/SnSe_2_^34^.

The highest peak current density observed in this study was achieved using a monolayer graphene, monolayer h-BN barrier RTT. The device was etched using the triangular etching approach to a graphene-overlapping area of *S* = 0.401 μm^2^, whereas the unetched corner was only *θ* = 8°, which could effectively suppress the effects of series and transmission-line resistances according to Eqs. (6)–(8). The room- and low-temperature output characteristics and peak current densities are shown in Fig. 4a–c. The peak current densities were up to 2.70 × 10^3^ and 3.60 × 10^3^ μA/μm^2^ at 300 and 9.8 K, respectively (with PVRs of 1.16 and 1.13, respectively). Two additional devices with high peak current densities are shown in Supplementary Figs. 10 and 11. The peak current density of a monolayer h-BN barrier RTT was up to 2.34 × 10^3^ μA/μm^2^ with a PVR of 1.45, and that of a bilayer h-BN barrier RTT was up to 1.25 × 10^3^ μA/μm^2^ with a PVR of 2.90.

The peak current density at low temperature, shown in Fig. 4c, increased by only 10–26% compared to that at room temperature under the same gate voltage, which may be due to series and transmission-line resistance limitations. To better understand the change in the peak current density with temperature, the electrical characteristics of a four-layer h-BN device at different temperatures were measured, as shown in Fig. 4d–f. A significant difference in the output characteristic curve at low temperature compared to that at room temperature is the appearance of a step (blue curves in Fig. 4e), which has been observed in the bilayer graphene RTT and is the result of the Fermi–Dirac distribution reflected in the output curve^18^. The peak current density at low temperature increased by 55–80% compared with that at room temperature, and the PVR also increased significantly.

The room-temperature peak current densities of RTTs fabricated with different numbers of graphene and h-BN barrier layers are shown in Fig. 4g. Bilayer graphene RTTs were also fabricated as detailed in the next section. The bilayer graphene RTT exhibited a peak current density comparable to that of the monolayer graphene RTT for the same number of h-BN barrier layers. The peak current densities for both monolayer and bilayer graphene increased exponentially with a decrease in the number of h-BN barrier layers. A comparison of the obtained results with those reported in the literature is shown in Fig. 4h.

### Effect of the number of graphene layers

In addition to the number of h-BN barrier layers, the effect of the number of graphene layers was also investigated. A bilayer graphene RTT was fabricated with a four-layer h-BN barrier to minimize the effects of series and transmission-line resistances. The output characteristics and PVR are shown in Supplementary Fig. 12. Unlike monolayer graphene RTTs, which have only one resonant tunneling condition, bilayer graphene RTTs have three tunneling conditions owing to the different energy band structure of bilayer graphene, thus resulting in double NDR characteristics with double peaks in each voltage polarity. We refer to the peak resulting from resonant tunneling between identical subbands as the near peak, and the two peaks resulting from resonant tunneling between different subbands as the far peaks. Bilayer graphene RTTs with trilayer and bilayer h-BN barriers were also fabricated as shown in Supplementary Figs. 13 and 14, respectively. The peak current densities were up to 865 μA/μm^2^. Double NDR is crucial to achieve three states in multivalued logic gates^36,39^. Bilayer graphene RTTs can also achieve a high current density by reducing the number of h-BN barrier layers and are promising for use in multivalued logic circuits with high switching speeds.

Combining literature reports and observations in this study, a feature position theory for monolayer and bilayer graphene RTTs was derived, as described in Supplementary Section 9. The monolayer graphene RTT has 2 noteworthy feature positions on the output characteristic curve, including 1 peak (Fig. 1c) and 1 step (Fig. 4e), whereas bilayer graphene RTT has 12 feature positions, including 3 peaks (Supplementary Fig. 12a), 5 steps (Supplementary Figs. 13, 16b, and 17b), and 4 intersections (Supplementary Fig. 12a, d).

### Toward high frequency

**Fig. 5:**
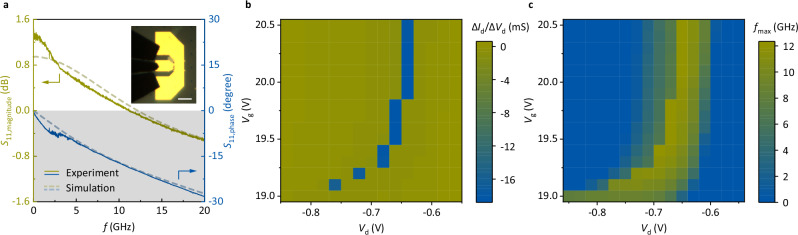
High-frequency operation of RTT. **a** Magnitude and phase of *S*_11_ as a function of the frequency *f* obtained in a high-frequency test for a monolayer graphene, bilayer h-BN barrier RTT, and their simulation results. The inset shows the optical micrograph of the device under test. The scale bar is 100 μm. **b**, **c** Mapping tests of the device along the gate voltage *V*_g_ and drain voltage *V*_d_, including the difference value Δ*I*_d_/Δ*V*_d_ in the d.c. test (**b**) and the maximum operating frequency *f*_max_ in the high-frequency test (**c**).

Peak current density is the key to the high-frequency operation of RTDs and RTTs. The maximum operating frequency *f*_max_ is expressed as^50^

10 $${f}_{\max }=\frac{G}{2\pi C}\sqrt{\frac{1}{G{R}_{{\rm{s}}}}-1}\approx \frac{d}{2\pi {\varepsilon }_{0}{\varepsilon }_{{\rm{r}}}}\frac{3\Delta J}{2\Delta V}\sqrt{\frac{1}{G{R}_{{\rm{s}}}}-1},$$

11 $$\Delta J={J}_{{{\rm{peak}}}}\frac{{{\rm{PVR}}}-1}{{{\rm{PVR}}}},$$

where *G* is the RTT tunneling conductance, *C* is the parasitic capacitance, *R*_s_ is the series resistance, *d* is the thickness of the h-BN barrier, *ε*_0_ is the vacuum dielectric constant, *ε*_r_ is the relative dielectric constant of h-BN, Δ*J* is the peak-to-valley current density difference, and Δ*V* is the peak-to-valley voltage difference. The maximum operating frequency is closely related to the peak current density and PVR. In this study, a monolayer graphene, bilayer h-BN barrier RTT with a coplanar waveguide (CPW) was fabricated on a high-resistivity silicon substrate. The device exhibited NDR characteristics in d.c. tests after etching to 2.97 μm^2^, as shown in Supplementary Fig. 18. With d.c. voltages (*V*_d_ = −0.65 V and *V*_g_ = 19.2 V) fixed in the NDR region, the s-parameter at an input-signal power of −15 dBm is shown in Fig. 5a. The relationship between the device impedance *Z*_RTT_ and *S*_11_ = | *S*_11_| e^i*φ*^ is given by

12 $${{Z}}_{{{\rm{RTT}}}}=\frac{1+{{S}}_{11}}{1-{{S}}_{11}}{{Z}}_{0}=\left(\frac{1-{\left|{{S}}_{11}\right|}^{2}}{1+{\left|{{S}}_{11}\right|}^{2}-2\left|{{S}}_{11}\right|\cos \varphi }+{\mbox{i}}\frac{2\left|{{S}}_{11}\right|\sin \varphi }{1+{\left|{{S}}_{11}\right|}^{2}-2\left|{{S}}_{11}\right|\cos \varphi }\right){{Z}}_{0},$$

where *Z*_0_ = 50 Ω. Equation (12) suggests that a necessary and sufficient condition for the real part of *Z*_RTT_ to be less than 0 is that the magnitude of *S*_11_ should be greater than 1 (dB value greater than 0). The experimental results shown in Fig. 5a demonstrate that the device maintained its NDR characteristics at frequencies up to 11 GHz. The simulation results obtained using High Frequency Structure Simulator (HFSS) software are also shown in Fig. 5a. The simulation estimated that the device had a negative tunneling resistance of approximately −1500 Ω, series resistance of approximately 600 Ω, and capacitance of approximately 7.9 fF (including the capacitance of the graphene/h-BN/graphene structure and the quantum capacitance of graphene). The effect of the transmission-line resistance was assumed to be effectively suppressed during etching and was not considered in the simulation. Furthermore, d.c. and high-frequency mapping tests along *V*_g_ and *V*_d_ were performed simultaneously, as shown in Fig. 5b, c. The NDR region (blue area in Fig. 5b) obtained in the d.c. test and the high maximum operating frequency region (yellow area in Fig. 5c) obtained in the high-frequency test agreed closely. The high-frequency performance of the trilayer h-BN barrier RTT was also tested, and a maximum operating frequency of only 73 MHz was obtained, as shown in Supplementary Fig. 19. This result suggests that current density is the key to high-frequency operations. The 11-GHz result obtained in this work is expected to support high-speed responses in 2D RTT applications such as multivalued logic gates and memory devices. Further etching of the device, optimization of the electrode design, thinning of the substrate, and replacing h-BN with a lower barrier 2D material could lead to much higher operating frequencies.

In summary, the effect of the number of graphene and h-BN atomic layers on the peak current densities of graphene/h-BN/graphene RTTs was investigated. For monolayer and bilayer h-BN barriers, high peak current densities resulted in the vanishing of the NDR characteristics. Modeling was performed to analyze the effects of different resistances in the circuit on the PVR and peak current density. Analytical calculations provided the effective transmission length of the tunneling current for the device design. The agreement between the experimental and theoretical results demonstrated that the graphene RTTs were affected by both series and transmission-line resistances. We were thereby able to propose a triangular etching approach which successfully suppressed the effects of both resistances and achieved pronounced NDR effect in a monolayer h-BN barrier RTT, which was not possible before. A high room-temperature peak current density of 2700 μA/μm^2^ was achieved. In the high-frequency test, the NDR performance was maintained at frequencies up to 11 GHz. This discovery fills the gap in the high-frequency operation of 2D NDR devices and has potential in future applications of 2D-materials-based electronics to achieve a high-speed response.

## Methods

### Device fabrication

Graphene and h-BN flakes were mechanically exfoliated from graphite crystals (NGS Naturgraphit) and h-BN crystals (2D Semiconductors) using an adhesive tape (Nitto ELP BT-130E-SL) onto Si/SiO_2_ substrates. Subsequently, the silicon wafer was placed under an optical microscope (Nikon ECLIPSE LV150N) to identify suitable graphene and h-BN flakes. The flakes were confirmed to be atomically flat with no wrinkles or steps inside and no contaminants attached using the dark-field mode of the microscope. The number of atomic layers of the flakes was determined using a dark-field optical contrast method developed by us^51^. The optical microscope was adjusted to dark-field mode, and a charge-coupled device camera was employed to capture dark-field images of the flake. The difference in brightness between the edge of the flake and the background was measured, and this relative brightness exhibited an approximately linear relationship with the flake thickness. Based on this relationship, the thickness of the flake could be determined with atomic-level precision.

A polydimethylsiloxane (PDMS) film was pasted onto the slide and polymethyl methacrylate (PMMA) was spin-coated onto the PDMS film. Subsequently, the slide was mounted on a 3D displacement stage that was manually operated under an optical microscope. h-BN, graphene, h-BN, graphene, and h-BN flakes were successively picked up using the PDMS/PMMA film to form a heterostructure, where a graphene flake was torn into two as the top and bottom graphene flakes. This technique is known as “tear and stack”^17^, and is used to provide the momentum matching required for resonant tunneling. The top and bottom graphene flakes were monolayers or bilayers, and the interlayer h-BN consisted of 1–6 layers. The top and bottom h-BN flakes were tens of nanometers thick and encapsulated the graphene/h-BN/graphene resonant tunneling structure to isolate it from air and improve device performance. The heterostructure was finally transferred onto a 675-μm-thick heavily p-doped silicon wafer with 300-nm-thick SiO_2_ for d.c. tests or onto a 200-μm-thick high-resistivity silicon wafer (>10,000 Ω cm) with 100-nm-thick SiO_2_ for high-frequency tests. The exfoliation and transfer processes were performed under ambient conditions.

Silicon was used as the gate electrode. The source and drain electrodes were defined using electron-beam lithography (EBL; Raith e-LiNE Plus). Graphene and h-BN were etched using ICP (Oxford Instruments PlasmaPro 100 Cobra) to expose the graphene edges. One-dimensional contacts^52^ were formed using Cr/Au, which were deposited via electron-beam evaporation (HHV Auto 500). For the d.c. tests, 3-nm-thick Cr and 70-nm-thick Au layers were deposited. For the high-frequency tests, the electrodes were designed in the shape of coplanar waveguides, and 3-nm Cr and 200-nm Au were deposited.

### Device characterization

The device was placed inside a cryogenic probe station (Lakeshore CRX-VF), and room- and low-temperature d.c. tests were performed using a source/measure unit (SMU; Keysight B2902A).

The device was placed on a high-frequency probe station, and high-frequency tests were performed using a vector network analyzer (VNA; Keysight N5247A PNA-X) equipped with a G–S–G probe. The SMU applies a d.c. gate voltage to the silicon substrate and a d.c. drain voltage via a bias tee to the VNA to enable the device to operate in the NDR region.

The device areas above 10 μm^2^ were measured using an optical microscope (Nikon ECLIPSE LV150N) with a charge coupled device (CCD), and those below 10 μm^2^ were measured using a scanning electron microscope (SEM; Nova NanoSEM 450) after the electrical test. Some SEM images are presented in Supplementary Fig. 20.

### Material characterization

The mechanically exfoliated h-BN samples were transferred from Si/SiO_2_ substrates onto molybdenum Quantifoil TEM grids. Bright-field scanning transmission electron microscopy (BF-STEM) images were collected using a JEOL ARM 200F instrument operating at 200 kV. A 40-μm condenser aperture was used as the probe-forming aperture, and a 3-mm BF-STEM aperture was used to improve the signal-to-noise ratio in the images. Images were acquired with an 8-cm camera length using a BF-STEM detector with an angular range of 0–17 mrad. The images show the hexagonal structure of h-BN along the [0001] zone axis.

## Supplementary information

Supplementary Information

Transparent Peer Review file

## Source data

Source Data

## Data Availability

The Source Data underlying the figures of this study are available with the paper. All raw data generated during the current study are available from the corresponding authors upon request. Source data are provided with this paper.
