# Supplementary material for: Toward high-current-density and high-frequency graphene resonant tunneling transistors
Source: Nat Commun. 2025 May 23;16:4805. doi: 10.1038/s41467-025-58720-7 (PMC12102250; doi:10.1038/s41467-025-58720-7)
Supplement: Supplementary file 1 — Supplementary Information [file 41467_2025_58720_MOESM1_ESM.pdf]

Supplementary Information of

Toward High-Current-Density and High-Frequency Graphene Resonant Tunneling Transistors

Zihao Zhang[1], Baoqing Zhang[1], Yifei Zhang[2], Yiming Wang[2], Patrick Hays[3], Seth Ariel Tongay[3], Mingyang Wang[2], Hecheng Han[2], Hu Li[2], Jiawei Zhang[2*], and Aimin Song[1,4*]

[1]Institute of Nanoscience and Applications, Southern University of Science and Technology, Shenzhen, 518055, China

[2]Shandong Technology Center of Nanodevices and Integration, School of Integrated Circuits, Shandong University, Jinan, 250100, China

[3]School for Engineering of Matter, Transport and Energy, Arizona State University, Tempe, AZ 85287, United States of America

[4]Department of Electrical and Electronic Engineering, University of Manchester, Manchester, M13 9PL, United Kingdom

[*]Correspondence to: Jiawei Zhang: Jiawei.Zhang@sdu.edu.cn
Aimin Song: songam@sustech.edu.cn

Table of Contents

Section 1 | Effect of the twist angle on RTTs with series resistance 4

Supplementary Figure 1 | Fabricated monolayer graphene, trilayer h-BN barrier RTTs with different twist angles and their series resistance deduction 4

Supplementary Figure 2 | Calculated results of the RTTs with different twist angles affected by series resistance 5

Supplementary Figure 3 | Output characteristics of a monolayer graphene, trilayer h-BN barrier RTT with a large twist angle of approximately 6°...6

Section 2 | Four-probe measurement...7

Supplementary Figure 4 | Two- and four-probe measurements of a monolayer graphene, trilayer h-BN barrier RTT affected by series resistance 7

Section 3 | Supplementary etching processes 8

Supplementary Figure 5 | Supplementary schematic and experimental results of the etching process of the bilayer h-BN barrier RTT in Fig. 1e..8

Supplementary Figure 6 | Rectangular and triangular etching control experiments 9

Supplementary Figure 7 | Rectangular and triangular etching control experiments with four-step etching...11

Section 4 | Numerical calculations of monolayer and bilayer graphene RTTs 12

Section 5 | Analytical calculations of the effect of the transmission-line resistance......................14

Supplementary Figure 8 | RTT cross-sectional schematic 14

Section 6 | h-BN characterization 17

Supplementary Figure 9 | STEM image of h-BN 17

Section 7 | Supplementary monolayer graphene RTTs with high peak current density.................18

Supplementary Figure 10 | Electrical characteristics of a monolayer graphene, monolayer h-BN barrier RTT 18

Supplementary Figure 11 | Electrical characteristics of a monolayer graphene, bilayer h-BN barrier RTT 18

Section 8 | Bilayer graphene RTTs...19

Supplementary Figure 12 | Electrical characteristics of a bilayer graphene, four-layer h-BN barrier RTT 19

Supplementary Figure 13 | Electrical characteristics of a bilayer graphene, trilayer h-BN barrier RTT 21

Supplementary Figure 14 | Electrical characteristics of a bilayer graphene, bilayer h-BN barrier RTT 21

Section 9 | Theoretical analyses of the feature positions of monolayer and bilayer graphene RTTs 22

Supplementary Figure 15 | Calculation results for the peak comparison of the monolayer and

bilayer graphene RTTs 23

Supplementary Figure 16 | Conditions of the peaks and steps of the monolayer and bilayer graphene RTTs 24

Supplementary Figure 17 | Calculation results for the step comparison of the monolayer and bilayer graphene RTTs 25

Supplementary Table 1 | Feature positions of monolayer and bilayer graphene RTTs 26

Section 10 | Supplementary information of high-frequency testing 27

Supplementary Figure 18 | Optical micrograph and d.c. output characteristics of the bilayer h-BN barrier RTT for high-frequency testing 27

Supplementary Figure 19 | D.c. and high-frequency test results of a trilayer h-BN barrier RTT 27

Section 11 | Area measurement of RTTs..28

Supplementary Figure 20 | Examples of RTT area measurement using SEM 28

Supplementary References 29

Section 1 | Effect of the twist angle on RTTs with series resistance

The device shown in Fig. 1d,g,h had two resonance peaks at both positive and negative voltages, indicating the presence of a large twist angle between the two graphene flakes. Two additional trilayer h-BN barrier devices with small and medium twist angles were subsequently fabricated, and the series resistance deduction was performed, as shown in Supplementary Fig. 1. The devices with different twist angles were affected differently by the series resistance. The distribution of the current peaks at different V_g values (dashed curves in Supplementary Fig. 1) was in the shape of "I" (Supplementary Fig. 1a), "J" (Supplementary Fig. 1c), or ">" (Fig. 1d) under the influence of series resistance, corresponding to a small, medium, or large twist angle, whereas the peaks without the influence of series resistance were distributed roughly in a straight line, regardless of the twist angle.

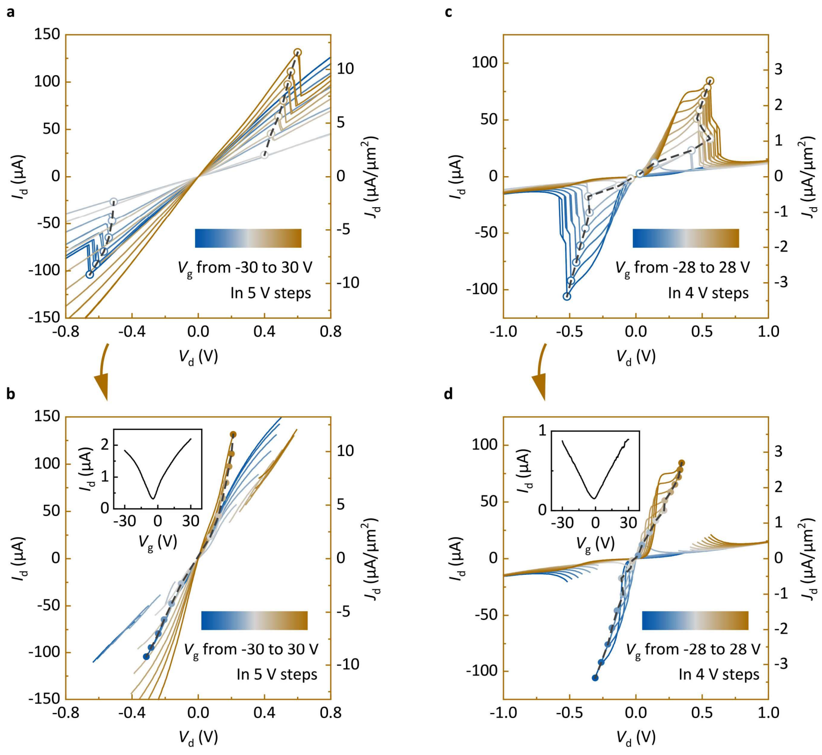

Supplementary Figure 1 | Fabricated monolayer graphene, trilayer h-BN barrier RTTs with different twist angles and their series resistance deduction. a–**d**, Measured output characteristics (**a**,**c**) and output characteristics with the series resistance deducted (**b**,**d**) of devices with small (**a**,**b**) and medium (**c**,**d**) twist angles. The insets of **b**,**d** show the transfer characteristics of graphene FETs, which were measured on one of the graphene flakes of the RTTs with small and medium twist angles, respectively, and used to deduct their series resistances.

This result was also confirmed by calculations, as shown in Supplementary Fig. 2. The numerical calculations described in Supplementary Section 4 were used, and the parameters were set as follows: $\gamma = 10$ meV; twist angle = 0°, 0.3°, and 1; $d_{SiO_2} = 300$ nm; $\varepsilon_{SiO_2} = 3.9$; $d_{hBN} = 1$ nm (for trilayer h-BN); $\varepsilon_{hBN} = 3.52$ (for trilayer h-BN; based on ref. [1]); $T = 300$ K. The "I"-, "J"-, and ">"-shaped peak distributions were obtained by setting the twist angle to 0°, 0.3°, and 1°, respectively. The

“>”-shaped peak distribution corresponding to a large twist angle is the most distinctive, and is therefore more suitable for demonstrating the effect of series resistance. In view of this, the large-twist-angle device is shown in Fig. 1d,g,h in the main text.

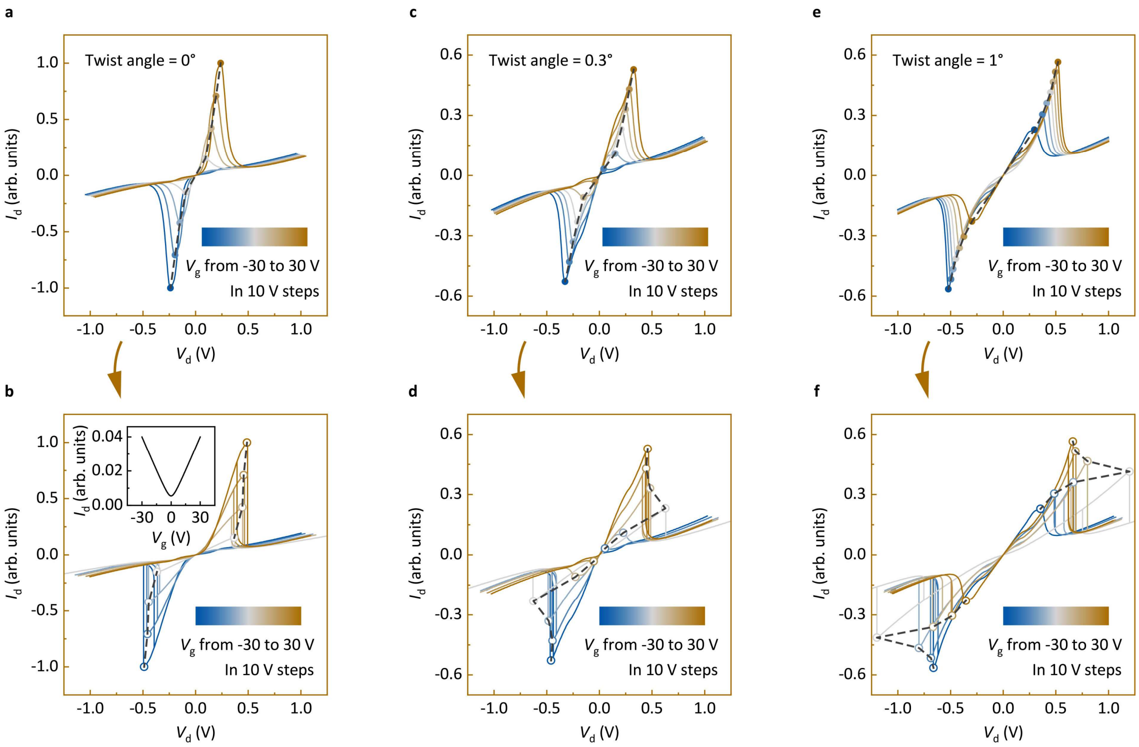

Supplementary Figure 2 | Calculated results of the RTTs with different twist angles affected by series resistance. a–f, Output characteristics without (**a**,**c**,**e**) and with (**b**,**d**,**f**) series resistance of devices with small (**a**,**b**), medium (**c**,**d**), and large (**e**,**f**) twist angles. The inset of **b** shows the calculated transfer characteristics of the graphene FET, which were used to calculate the results in **b**,**d**,**f**.

A device with a larger twist angle of approximately 6° was fabricated, as shown in Supplementary Fig. 3. The resonance peaks were located at higher voltages and were less modulated by the gate voltage. Combined with Fig. 1h and Supplementary Figs. 1b, 1d, and 3, the peak current density was not found to be significantly correlated with the twist angle.

Note that none of the twist angles of the bilayer and monolayer h-BN barrier devices in this study were approximately 0°. There are two possible reasons for this result. First, the thermal effect from the high peak current density may rotate the graphene relative to the h-BN, as has been reported in the literature[2]. Second, the small areas of the bilayer and monolayer h-BN barrier devices facilitate thermally induced rotation.

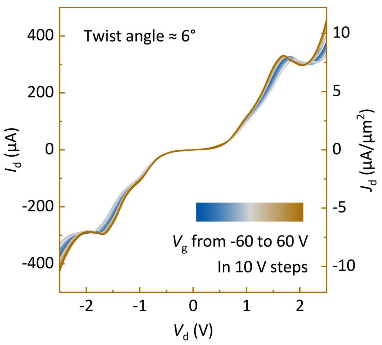

Supplementary Figure 3 | Output characteristics of a monolayer graphene, trilayer h-BN barrier RTT with a large twist angle of approximately 6°.

Section 2 | Four-probe measurement

The four-probe measurement of the trilayer h-BN barrier device is shown in Supplementary Fig. 4. The inset of Supplementary Fig. 4b shows a lower voltage at the current peak in the four-probe measurement compared to the two-probe measurement, owing to the deduction of the graphene–metal contact resistance in the four-probe measurement[3]. However, because none of the four electrodes could be infinitely close to the graphene-overlapping region, the device was still affected by the resistance of the graphene in series. Additionally, the introduction of a constant current source resulted in the loss of information in the NDR region, but provided a simple platform to demonstrate the memory application of RTT[4].

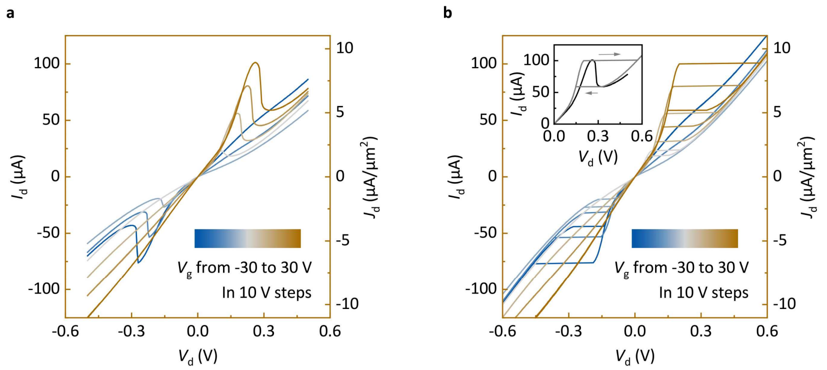

Supplementary Figure 4 | Two- and four-probe measurements of a monolayer graphene, trilayer h-BN barrier RTT affected by series resistance. **a**, Two-probe measurement results. **b**, Four-probe measurement results. The inset is a comparison of two-probe (black) and four-probe (gray) measurements at V_g = 30 V. The arrows indicate forward and backward sweeps.

Section 3 | Supplementary etching processes

Supplementary Fig. 5a shows a schematic of the etching of the bilayer h-BN barrier device in Fig. 1e. Supplementary Fig. 5b–e shows the etching process for another part of the device using the triangular etching approach. During the two-step etching, NDR was observed at more V_g values, and the peak current density increased, which agrees with Fig. 2c–f. The device areas were S = 8.17 μm^2 and S = 3.81 μm^2 after the first and second etching steps, respectively.

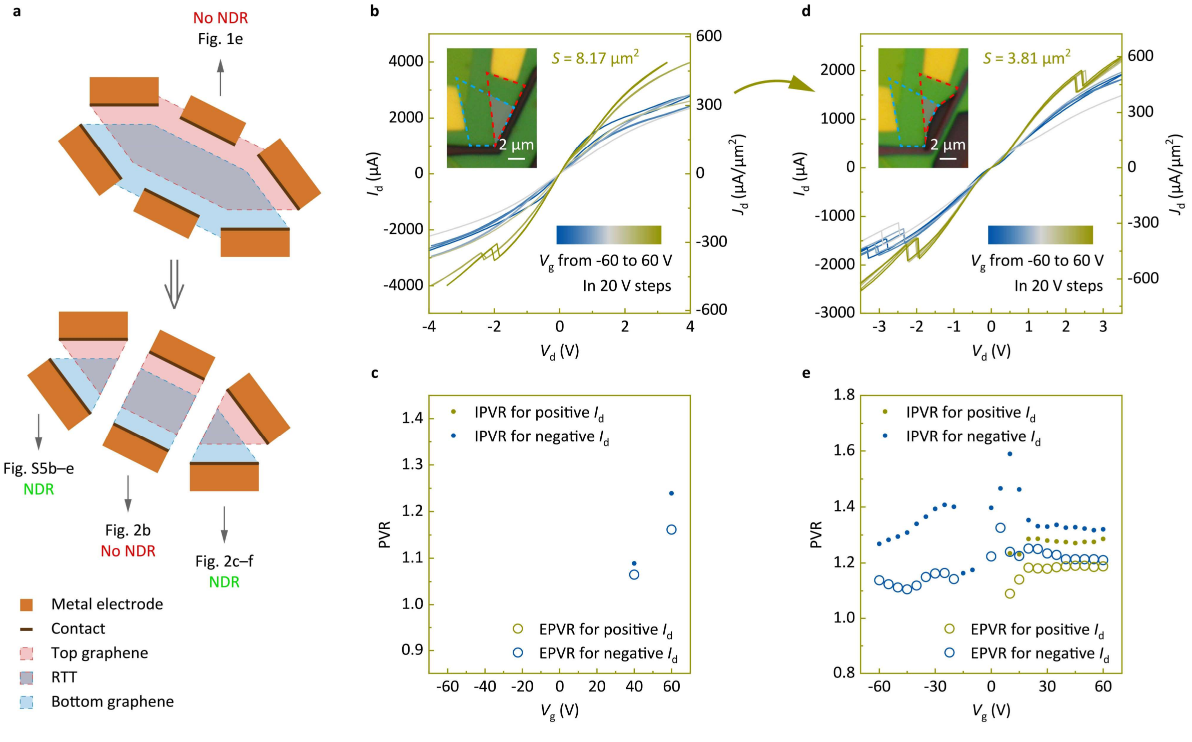

Supplementary Figure 5 | Supplementary schematic and experimental results of the etching process of the bilayer h-BN barrier RTT in Fig. 1e. a, Etching schematic. **b**–**e**, Output characteristics (**b**,**d**) and PVRs (**c**,**e**) of the device after the first (**b**,**c**) and second (**d**,**e**) etching steps using the triangular etching approach. The insets of **b**,**d** show optical micrographs of the device after the corresponding etching step. The red and blue dashed lines indicate the ranges of the top and bottom graphene flakes, respectively.

An additional bilayer h-BN barrier RTT was fabricated, and a set of rectangular and triangular etching control experiments was performed, as shown in Supplementary Fig. 6. In this set of experiments, the rectangularly etched subdevice was slightly smaller than the triangularly etched subdevice. However, the latter exhibited obvious NDR characteristics, whereas the former did not, in agreement with the model considering the series and transmission-line resistances.

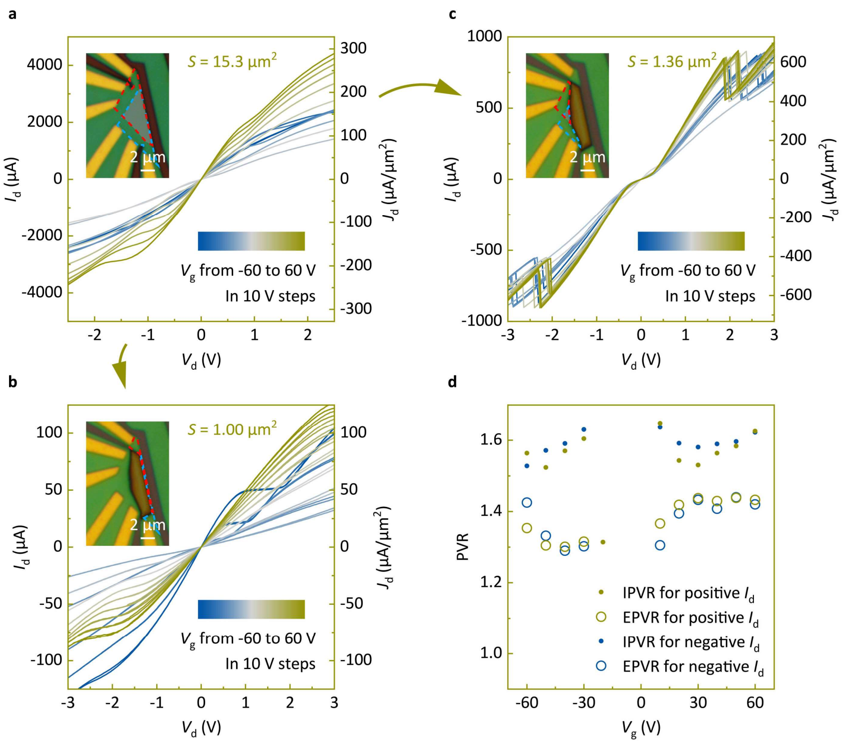

Supplementary Figure 6 | Rectangular and triangular etching control experiments. a, Output characteristics of a monolayer graphene, bilayer h-BN barrier RTT before etching. **b**, Output characteristics of a part of the device after rectangular etching. **c**,**d**, Output characteristics (**c**) and PVR (**d**) of the other part of the device after triangular etching. The insets of **a**–**c** show optical micrographs of the device before etching and after the corresponding etching step. The red and blue dashed lines indicate the ranges of the top and bottom graphene flakes, respectively.

Another bilayer h-BN barrier RTT was fabricated and etched four times to investigate the variations in PVR and peak current density with etching, as shown in Supplementary Fig. 7. After the device was cut into two parts, one part was rectangularly etched and the other part was triangularly etched. The PVR and peak current density of the triangularly etched subdevice gradually increased, whereas the rectangularly etched subdevice exhibited no NDR characteristics from the beginning to the end. The variation in the PVR shown in Supplementary Fig. 7b agrees closely with the theoretical analyses shown in Fig. 3b,e.

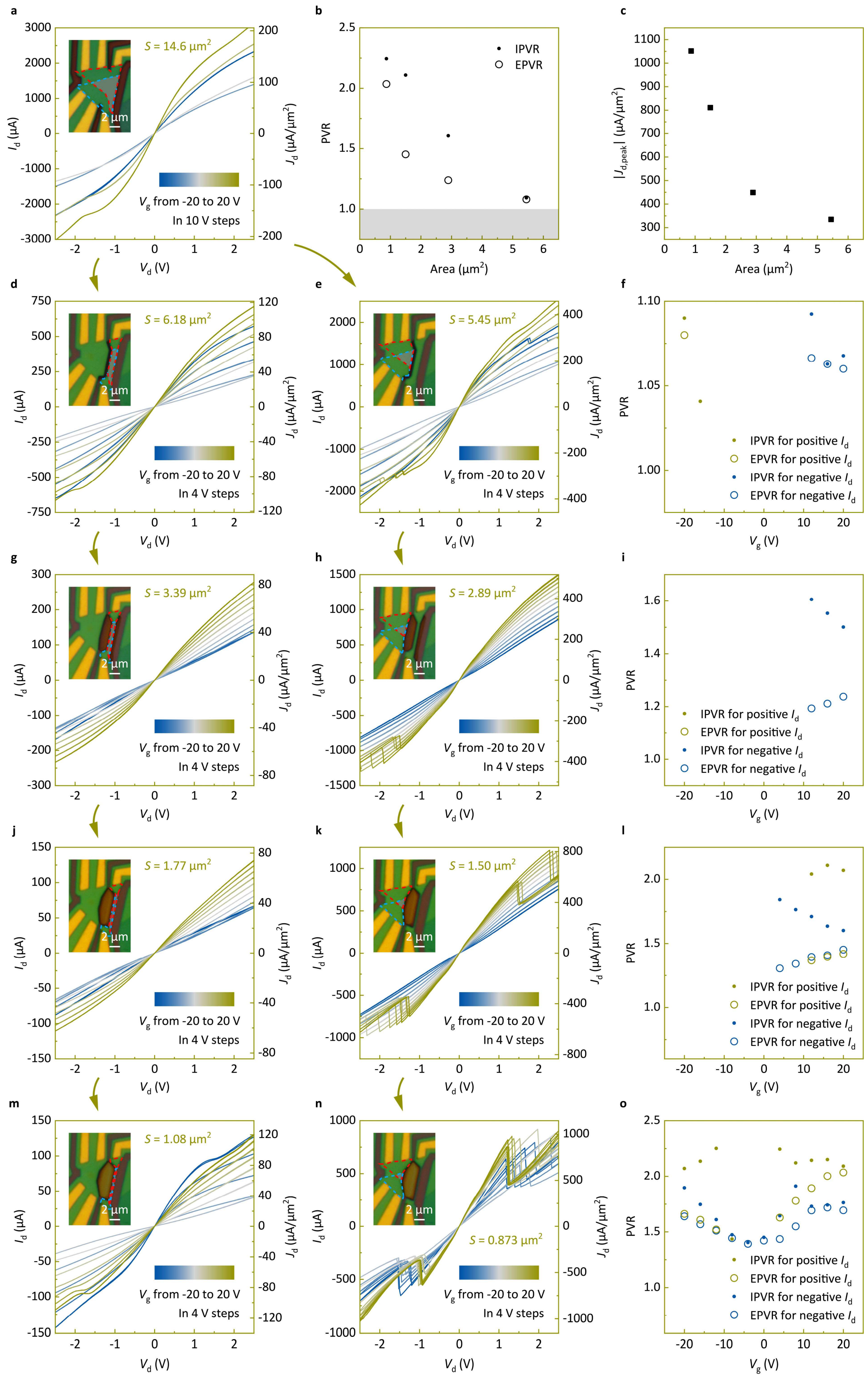

a
S = 14.6 μm2
2 μm
Vg from -20 to 20 V
In 10 V steps
Id (μA)
Jd (μA/μm2)
Vd (V)
b
IPVR
EPVR
PVR
Area (μm2)
c
|Jd,peak| (μA/μm2)
Area (μm2)
d
S = 6.18 μm2
Vg from -20 to 20 V
In 4 V steps
e
S = 5.45 μm2
Vg from -20 to 20 V
In 4 V steps
f
IPVR for positive Id
EPVR for positive Id
IPVR for negative Id
EPVR for negative Id
Vg (V)
g
S = 3.39 μm2
Vg from -20 to 20 V
In 4 V steps
h
S = 2.89 μm2
Vg from -20 to 20 V
In 4 V steps
i
IPVR for positive Id
EPVR for positive Id
IPVR for negative Id
EPVR for negative Id
j
S = 1.77 μm2
Vg from -20 to 20 V
In 4 V steps
k
S = 1.50 μm2
Vg from -20 to 20 V
In 4 V steps
l
IPVR for positive Id
EPVR for positive Id
IPVR for negative Id
EPVR for negative Id
m
S = 1.08 μm2
Vg from -20 to 20 V
In 4 V steps
n
S = 0.873 μm2
Vg from -20 to 20 V
In 4 V steps
o
IPVR for positive Id
EPVR for positive Id
IPVR for negative Id
EPVR for negative Id

Supplementary Figure 7 | Rectangular and triangular etching control experiments with four-step etching. a, Output characteristics of a monolayer graphene, bilayer h-BN barrier RTT before etching. **b**,**c**, Variation of the highest PVR (**b**) and highest peak current density (**c**) with area during the four-step triangular etching. **d**–**o**, Output characteristics of the part of the device with rectangular etching (**d**,**g**,**j**,**m**), output characteristics of the part with triangular etching (**e**,**h**,**k**,**n**), and PVR of the part with triangular etching (**f**,**i**,**l**,**o**) after the first (**d**,**e**,**f**), second (**g**,**h**,**i**), third (**j**,**k**,**l**), and fourth (**m**,**n**,**o**) etching steps. The insets show optical micrographs of the device before etching and after the corresponding etching step. The red and blue dashed lines indicate the ranges of the top and bottom graphene flakes, respectively.

Section 4 | Numerical calculations of monolayer and bilayer graphene RTTs

Theoretical calculations of monolayer and bilayer graphene RTTs have been reported in the literature[3,5–8]. On the basis of these previous studies, the initial *J*–*V* or *I*–*V* curves were numerically calculated in this study to further evaluate the effects of the series and transmission-line resistances shown in Fig. 3 and Supplementary Fig. 2, and the feature positions of the monolayer and bilayer graphene RTTs were compared as discussed in Supplementary Section 9. The series and transmission-line resistances were ignored in this section. The energy E_{MG} on the monolayer graphene band and E_{BG} on the Bernal-stacked bilayer graphene band are related to the wave vector k in reciprocal space as

$$E_{MG}\left(k\right) = s\hbar v_F k, \tag{1}$$

$$E_{BG}\left(k\right) = s\left(\mu\frac{\gamma_1}{2} + \sqrt{\frac{\gamma_1^2}{4} + \left(\hbar v_F k\right)^2}\right), \tag{2}$$

where $s = \pm 1$, $\mu = \pm 1$, $\gamma_1 = 0.39$ eV, and v_F is the Fermi velocity of monolayer graphene. The sheet carrier density n_{MG} of the monolayer graphene and n_{BG} of the bilayer graphene (negative for electrons and positive for holes) as a function of the Fermi level E_F is given by

$$n_{MG}\left(E_F\right) = \begin{cases} \dfrac{E_F^2}{\pi\hbar^2 v_F^2}, & E_F < 0 \\ -\dfrac{E_F^2}{\pi\hbar^2 v_F^2}, & E_F \geq 0 \end{cases}, \tag{3}$$

$$n_{BG}\left(E_F\right) = \begin{cases} \dfrac{2E_F^2}{\pi\hbar^2 v_F^2}, & E_F < -\gamma_1 \\ \dfrac{E_F^2 - \gamma_1 E_F}{\pi\hbar^2 v_F^2}, & -\gamma_1 \leq E_F < 0 \\ -\dfrac{E_F^2 + \gamma_1 E_F}{\pi\hbar^2 v_F^2}, & 0 \leq E_F < \gamma_1 \\ -\dfrac{2E_F^2}{\pi\hbar^2 v_F^2}, & E_F \geq \gamma_1 \end{cases}. \tag{4}$$

The tunneling current of graphene RTT is expressed as

$$I_d \propto \iint \frac{\gamma}{\left(E_T - E_B + \Delta\varphi\right)^2 + \gamma^2}\left(f_T - f_B\right) dk_x dk_y, \tag{5}$$

where $k_{x/y}$ is the wave vector in the x or y direction in reciprocal space, $E_{T/B}$ is the energy of the top or bottom graphene flake, $\Delta\varphi$ is the energy difference of the Dirac point from the top to the bottom graphene flake, γ is the broadening of electronic states, and $f_{T/B}$ is the Fermi–Dirac distribution and is expressed as

$$f_{T/B} = \frac{1}{e^{\frac{E_{T/B} - E_{F,T/B}}{k_{Bol}T}} + 1}, \tag{6}$$

where $E_{F,T/B}$ is the Fermi level of the top or bottom graphene flake, k_{Bol} is Boltzmann constant, and T is the temperature. Considering the balance of potential energy, we obtain

$$E_{F,T} - E_{F,B} + \Delta\varphi - eV_d = 0, \tag{7}$$

where V_d denotes the bias voltage applied to the device. Considering the conservation of charge,

we obtain

$$n_{\mathrm{T}} + n_{\mathrm{B}} + n_{\mathrm{g}} = 0, \tag{8}$$

where $n_{\mathrm{T/B/g}}$ is the sheet carrier density of the top graphene flake, bottom graphene flake, or silicon gate, and satisfies

$$eV_{\mathrm{g}} = \frac{e^2 d_{\mathrm{SiO_2}} n_{\mathrm{g}}}{\varepsilon_0 \varepsilon_{\mathrm{SiO_2}}}, \tag{9}$$

$$\Delta\varphi = -\frac{e^2 d_{\mathrm{hBN}} n_{\mathrm{T}}}{\varepsilon_0 \varepsilon_{\mathrm{hBN}}}, \tag{10}$$

where V_{g} is the gate voltage, $d_{\mathrm{SiO_2}}$ is the thickness of the SiO_2 gate dielectric, $\varepsilon_{\mathrm{SiO_2}}$ is the relative dielectric constant of SiO_2, d_{hBN} is the thickness of the h-BN barrier, and $\varepsilon_{\mathrm{hBN}}$ is the relative dielectric constant of h-BN. The results of the calculation can be obtained by substituting Supplementary equations (6)–(10) into Supplementary equation (5) and substituting Supplementary equations (1) and (3) for monolayer graphene or Supplementary equations (2) and (4) for bilayer graphene into Supplementary equation (5). The integral traverses the linear bands of monolayer graphene or the approximately parabolic bands of bilayer graphene.

Section 5 | Analytical calculations of the effect of the transmission-line resistance

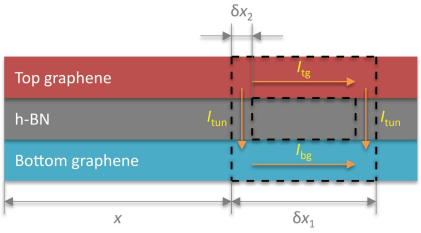

Supplementary Figure 8 | RTT cross-sectional schematic.

Numerical calculations have been provided in Fig. 3 in the main text, and analytical calculations of the device characteristics affected by the transmission-line resistance were performed as described here. The analytical calculations required fixing the resistances to a constant value that did not vary with voltage and spatial location, and ignoring the quantum capacitance of graphene, which lost some accuracy but helped in observing more mechanisms, such as how inhomogeneous current and bias distributions in the device caused peak current density loss. A cross-sectional schematic of the RTT is shown in Supplementary Fig. 8, where L is the device length, D is the device width, r_{tg} is the top graphene sheet resistance, r_{bg} is the bottom graphene sheet resistance, r_{tun} is the tunneling resistance per unit area, I_d is the drain current, $I_{tg}(x)$ is the top graphene current at spatial location x along the length of the device, $I_{bg}(x)$ is the bottom graphene current, and $I_{tun}(x)$ is the tunneling current per unit length. By examining a loop in a small area of space, where $\delta x_2 \ll \delta x_1 \ll L$, according to Kirchhoff's law, we obtain

$$I_{tg}(x) + I_{bg}(x) = I_d, \tag{11}$$

$$I_{tun}(x) = \frac{d}{dx} I_{bg}(x), \tag{12}$$

$$I_{tg}(x)\frac{r_{tg}\delta x_1}{D} + I_{tun}(x+\delta x_1)\,\delta x_2\frac{r_{tun}}{D\delta x_2} - I_{bg}(x)\frac{r_{bg}\delta x_1}{D} - I_{tun}(x)\,\delta x_2\frac{r_{tun}}{D\delta x_2} = 0. \tag{13}$$

If the current enters the top graphene flake from the left side and leaves the bottom graphene flake from the right side, we obtain

$$I_{tg}(0) = I_d, \tag{14}$$

$$I_{bg}(L) = I_d. \tag{15}$$

The solutions to these equations are

$$\begin{aligned} I_{tg}(x) = {} & I_d \frac{r_{tg} e^{\sqrt{\frac{r_{tg}+r_{bg}}{r_{tun}}}L} + r_{bg}}{2\left(r_{tg}+r_{bg}\right)\sinh\sqrt{\frac{r_{tg}+r_{bg}}{r_{tun}}}L} e^{-\sqrt{\frac{r_{tg}+r_{bg}}{r_{tun}}}x} + I_d\frac{r_{bg}}{r_{tg}+r_{bg}} \\ & - I_d \frac{r_{tg} + r_{bg} e^{\sqrt{\frac{r_{tg}+r_{bg}}{r_{tun}}}L}}{2\left(r_{tg}+r_{bg}\right)\sinh\sqrt{\frac{r_{tg}+r_{bg}}{r_{tun}}}L} e^{-\sqrt{\frac{r_{tg}+r_{bg}}{r_{tun}}}(L-x)}, \end{aligned} \tag{16}$$

$$I_{bg}(x) = -I_d \frac{r_{tg} e^{\sqrt{\frac{r_{tg}+r_{bg}}{r_{tun}}}L} + r_{bg}}{2\left(r_{tg}+r_{bg}\right)\sinh\sqrt{\frac{r_{tg}+r_{bg}}{r_{tun}}}L} e^{-\sqrt{\frac{r_{tg}+r_{bg}}{r_{tun}}}x} + I_d \frac{r_{tg}}{r_{tg}+r_{bg}}$$

$$+ I_d \frac{r_{tg} + r_{bg} e^{\sqrt{\frac{r_{tg}+r_{bg}}{r_{tun}}}L}}{2\left(r_{tg}+r_{bg}\right)\sinh\sqrt{\frac{r_{tg}+r_{bg}}{r_{tun}}}L} e^{-\sqrt{\frac{r_{tg}+r_{bg}}{r_{tun}}}(L-x)}, \tag{17}$$

$$I_{tun}(x) = I_d \frac{r_{tg} e^{\sqrt{\frac{r_{tg}+r_{bg}}{r_{tun}}}L} + r_{bg}}{2\left(r_{tg}+r_{bg}\right)\sinh\sqrt{\frac{r_{tg}+r_{bg}}{r_{tun}}}L} \sqrt{\frac{r_{tg}+r_{bg}}{r_{tun}}} e^{-\sqrt{\frac{r_{tg}+r_{bg}}{r_{tun}}}x}$$

$$+ I_d \frac{r_{tg} + r_{bg} e^{\sqrt{\frac{r_{tg}+r_{bg}}{r_{tun}}}L}}{2\left(r_{tg}+r_{bg}\right)\sinh\sqrt{\frac{r_{tg}+r_{bg}}{r_{tun}}}L} \sqrt{\frac{r_{tg}+r_{bg}}{r_{tun}}} e^{-\sqrt{\frac{r_{tg}+r_{bg}}{r_{tun}}}(L-x)}. \tag{18}$$

The first term in Supplementary equation (18) indicates a rightward exponentially decaying current component near the left edge of the device, and the second term indicates a leftward exponentially decaying current component near the right edge. When $(r_{tg} + r_{bg})\, L \gg r_{tun}L^{-1}$, the tunneling current is concentrated in a range of approximately $\sqrt{r_{tun}/\left(r_{tg}+r_{bg}\right)}$ near the left and right edges, with almost no tunneling current in the middle. The ratio of the left- and right-side tunneling currents is equal to the ratio of the top and bottom transmission-line resistances, r_{tg}/r_{bg}. When $r_{tg/bg} = 0$, the tunneling current is concentrated only near the right or left edge. When $(r_{tg} + r_{bg})\, L \ll r_{tun}L^{-1}$, the tunneling current is distributed almost homogeneously. The inhomogeneities of the distributions of the tunneling current and the bias $V_{tun}(x) = I_{tun}(x)\, r_{tun}/D$ increase with $(r_{tg} + r_{bg})\, L/r_{tun}L^{-1}$. Therefore, the transmission-line resistance caused the inhomogeneity of the bias and disrupted the consistency of the resonant tunneling conditions at different x values, resulting in the broadening and depression of the resonant tunneling peaks and reduction of the IPVR and peak current density of the device, whereas the series resistance did not have these effects. Moreover, the inhomogeneity of the tunneling current reduced the effective device area, also resulting in a decrease in the peak current density.

The drain voltage is expressed as

$$V_d = \int_0^L I_{tg}(x) \frac{r_{tg}\mathrm{d}x}{D} + \left(I_{tun}(x)\, \mathrm{d}x \frac{r_{tun}}{D\mathrm{d}x}\right)\Big|_L$$

$$= I_d \frac{r_{tg}r_{bg}}{r_{tg}+r_{bg}} \frac{L}{D} + I_d \frac{\sqrt{r_{tun}}}{D} \frac{\left(r_{tg}^2 + r_{bg}^2\right)\cosh\sqrt{\frac{r_{tg}+r_{bg}}{r_{tun}}}L + 2r_{tg}r_{bg}}{\left(r_{tg}+r_{bg}\right)^{\frac{3}{2}}\sinh\sqrt{\frac{r_{tg}+r_{bg}}{r_{tun}}}L}. \tag{19}$$

When $(r_{tg} + r_{bg})\, L \gg r_{tun}L^{-1}$, we obtain

$$V_d = I_d \frac{r_{tg}r_{bg}}{r_{tg}+r_{bg}} \frac{L}{D} + I_d \frac{\sqrt{r_{tun}}}{D} \frac{r_{tg}^2 + r_{bg}^2}{\left(r_{tg}+r_{bg}\right)^{\frac{3}{2}}}. \tag{20}$$

If $r_{tg} > 0$ and $r_{bg} > 0$, we obtain

$$V_d = I_d \frac{r_{tg} r_{bg}}{r_{tg} + r_{bg}} \frac{L}{D}, \tag{21}$$

which is equivalent to r_{tg} and r_{bg} in parallel, and r_{tun} does not operate in the circuit. If $r_{bg/tg} = 0$, we obtain

$$V_d = I_d \frac{\sqrt{r_{tun} r_{tg/bg}}}{D}, \tag{22}$$

which is independent of L because the tunneling current is concentrated only near the left or right edge. When $(r_{tg} + r_{bg})\, L \ll r_{tun} L^{-1}$, we obtain

$$V_d = I_d \frac{r_{tun}}{LD} = J_d r_{tun}, \tag{23}$$

indicating that r_{tg} and r_{bg} do not play a role and that only ideal resonant tunneling exists in the circuit. The case shown in Supplementary equation (23) can be approached using the triangular etching approach.

Section 6 | h-BN characterization

Supplementary Fig. 9 shows one of the STEM images of h-BN used in this study, which indicates its lattice integrity.

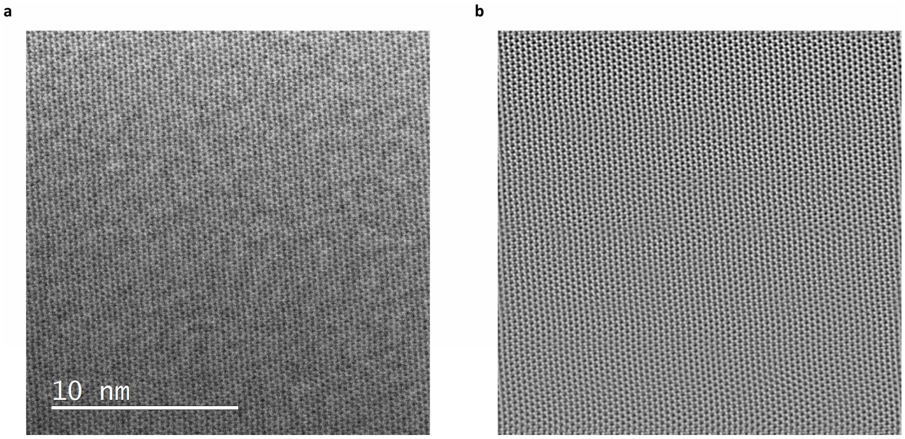

Supplementary Figure 9 | STEM image of h-BN. a, Raw image. **b**, Fast Fourier transform filtered image of **a**.

Section 7 | Supplementary monolayer graphene RTTs with high peak current density

A monolayer graphene, monolayer h-BN barrier device was fabricated and etched using the triangular etching approach to a device area of S = 0.477 μm^2, whereas the unetched corner was ϑ = 16°. The peak current density was up to 2.34 × 10^3 μA/μm^2 with a PVR of 1.45, as shown in Supplementary Fig. 10.

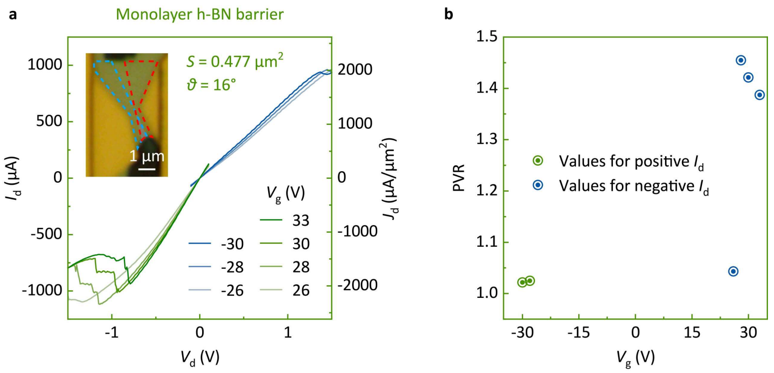

Supplementary Figure 10 | Electrical characteristics of a monolayer graphene, monolayer h-BN barrier RTT. a, Output characteristics. The inset shows an optical micrograph of the device. The red and blue dashed lines indicate the ranges of the top and bottom graphene flakes, respectively. **b**, PVR.

A monolayer graphene, bilayer h-BN barrier device was fabricated and etched using the triangular etching approach to a device area of S = 0.685 μm^2. The peak current density was up to 1.25 × 10^3 μA/μm^2 with a PVR of 2.90, as shown in Supplementary Fig. 11.

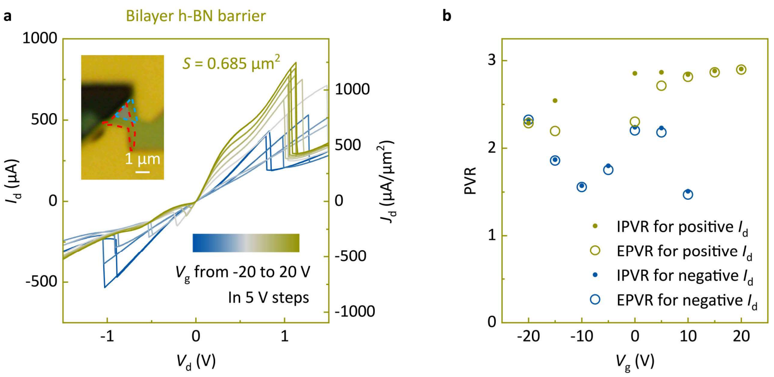

Supplementary Figure 11 | Electrical characteristics of a monolayer graphene, bilayer h-BN barrier RTT. a, Output characteristics. The inset shows an optical micrograph of the device. The red and blue dashed lines indicate the ranges of the top and bottom graphene flakes, respectively. **b**, PVR.

Section 8 | Bilayer graphene RTTs

A bilayer graphene, four-layer h-BN barrier device was fabricated. The output characteristics and PVR are shown in Supplementary Fig. 12a,b. As shown in Supplementary Fig. 12a, the curves of different V_g values converged to a point at four positions. Supplementary Fig. 12c shows a plot of the on/off ratio of the device in the range of V_g from −120 to 120 V. It can be seen that the on/off ratio is nearly 1 at the four values of V_d = −1.05, −0.34, 0.33, and 1.02 V. Supplementary Fig. 12d shows a plot of the transfer characteristics at these four V_d values. The current of the device was modulated only by 4%–8% in the V_g range of 120 V. We refer to these four positions that converge the output characteristic curves and flatten the transfer characteristic curves as the intersections of the bilayer graphene RTT, where the two intersections between the near and far peaks are referred to as the near intersections, and the two intersections outside the far peaks are referred to as the far intersections.

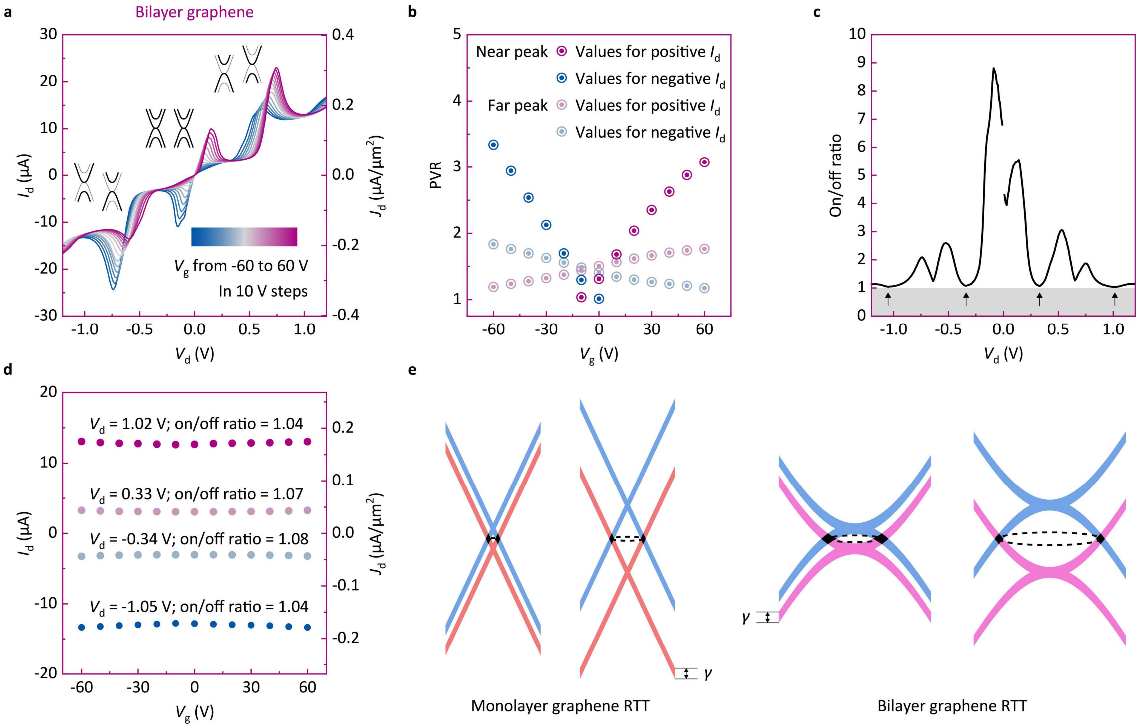

Supplementary Figure 12 | Electrical characteristics of a bilayer graphene, four-layer h-BN barrier RTT. a,**b**, Output characteristics (**a**) and PVR (**b**), in which the drain current I_d and drain current density J_d change with the drain voltage V_d under different gate voltages V_g. The insets of **a** next to the curves show the different energy band matching modes of Bernal-stacked bilayer graphene corresponding to each resonant tunneling peak. **c**, On/off ratio as a function of V_d. The arrows indicate four positions where the on/off ratio approaches 1. **d**, Transfer characteristics under the four V_d values with the on/off ratio approaching 1. **e**, Schematic of monolayer and bilayer graphene interband tunneling. The bands of the top graphene are indicated in red or purple, and the bands of the bottom graphene are indicated in blue. For bilayer graphene, interband tunneling between only one pair of subbands is presented.

The cause of the intersections of the bilayer graphene RTT was analyzed. Graphene has a zero-state density at the Dirac point, but empirically it has a nonzero minimum conductivity even at extremely low temperatures owing to disorder[9]. The disorder of graphene is experimentally manifested as electron–hole puddles[9] and can be theoretically described by a phenomenological parameter, the broadening of electronic states γ (refs. [6,10]). A physical model was established, as shown in Supplementary Fig. 12e. Here, the bands of graphene were assumed to have a thickness of γ in the energy direction, and for bilayer graphene, only one pair of subbands were considered. Thus, the band expressions of monolayer and bilayer graphene are rewritten from Supplementary equations (1) and (2) as follows, where bilayer graphene has an approximate quadratic dispersion relation near the Dirac point:

$$E_{\mathrm{MG}}\,(k) \in \left(s\hbar v_{\mathrm{F}}k - \frac{\gamma}{2},\, s\hbar v_{\mathrm{F}}k + \frac{\gamma}{2}\right), \tag{24}$$

$$E_{\mathrm{BG}}\,(k) \in \left(s\left(\frac{\left(\hbar v_{\mathrm{F}}k\right)^2}{\gamma_1} - \frac{\gamma}{2}\right),\, s\left(\frac{\left(\hbar v_{\mathrm{F}}k\right)^2}{\gamma_1} + \frac{\gamma}{2}\right)\right). \tag{25}$$

Interband tunneling of graphene occurs in the overlapping region of the vertically misaligned bands of the two graphene flakes. The overlapping region is approximated as a torus with a rhombic cross section. The interband tunneling currents of monolayer and bilayer graphene RTTs are proportional to the volume of the overlapped region, and are thereby expressed in terms of the momentum k of the overlapped region as

$$I_{\mathrm{MG,interband}}\,(k) \propto 2\pi k\cdot\frac{\Delta k \Delta E_{\mathrm{MG}}}{2} = \frac{\pi\gamma^2}{\hbar v_{\mathrm{F}}}k \propto k, \tag{26}$$

$$I_{\mathrm{BG,interband}}\,(k) \propto 2\pi k\cdot\frac{\Delta k \Delta E_{\mathrm{BG}}}{2} = \frac{\pi\gamma^2\gamma_1}{2\hbar^2 v_{\mathrm{F}}^2} = \text{constant}, \tag{27}$$

where Δk and $\Delta E_{\mathrm{MG/BG}}$ are the lengths of the two diagonal lines of the rhombus. Clearly, the interband tunneling current of the monolayer graphene RTT is proportional to the vertical misalignment distance of the graphene bands, whereas that of the bilayer graphene RTT is independent of this distance. This result implies that the interband tunneling current of the bilayer graphene RTT cannot be modulated in the ideal case assumed by the above model. Theoretically, the resonant tunneling peaks of the bilayer graphene RTT are mainly attributed to intraband tunneling, and the valleys are mainly attributed to interband tunneling. Therefore, the unmodulatability of interband tunneling is reflected in the electrical characteristics, in which the tunneling current remains constant around the four resonant tunneling valleys of the bilayer graphene RTT for a certain interval of V_{d} and V_{g}. The experimental results demonstrate that this property is more pronounced for V_{g} modulation, which can modulate the tunneling current only by 4% in a wide V_{g} range of 120 V.

A bilayer graphene, trilayer h-BN barrier RTT was fabricated, and a comparison of the room- and low-temperature output characteristics is shown in Supplementary Fig. 13. At low temperature as shown in Supplementary Fig. 13b, in addition to the three resonance peaks, three step-like features were observed in the curve (inside the dashed circles). One is near $V_d = 0$ V, which is always in the opposite voltage polarity of the near peak, and we refer to it as the near step. Two are near $V_d = \pm 1.3$ V, and we refer to them as the far steps. The near step has been reported in the literature: Reference [6] demonstrated the near step in a device with a twist angle of graphene, and ref. [3] discussed the temperature dependence of the near step. As shown in Supplementary Fig. 13a, the near step is almost invisible at room temperature, whereas the visibility of the far steps is not much affected by temperature.

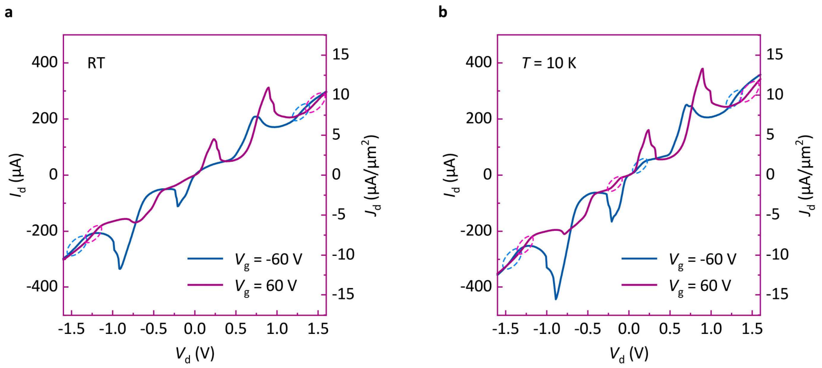

Supplementary Figure 13 | Electrical characteristics of a bilayer graphene, trilayer h-BN barrier RTT. a, At room temperature. **b**, At 10 K.

A bilayer graphene, bilayer h-BN barrier device was fabricated. The peak current density was up to 865 $\mu A/\mu m^2$ after etching to a device area of $S = 3.02\ \mu m^2$, as shown in Supplementary Fig. 14.

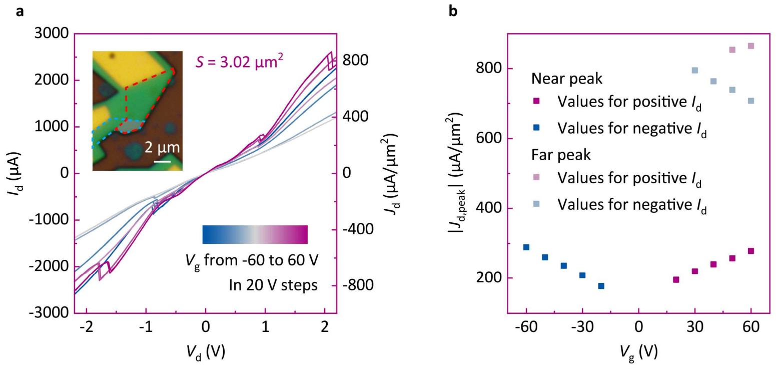

Supplementary Figure 14 | Electrical characteristics of a bilayer graphene, bilayer h-BN barrier RTT. a, Output characteristics. The inset shows an optical micrograph of the device. The red and blue dashed lines indicate the ranges of the top and bottom graphene flakes, respectively. **b**, Peak current density.

Section 9 | Theoretical analyses of the feature positions of monolayer and bilayer graphene RTTs

We collectively refer to the peaks, steps, and intersections of graphene RTTs observed in this study as feature positions. The theoretical analyses of the intersections have been presented in Supplementary Fig. 12. In this section, the peaks and steps of the monolayer and bilayer graphene RTTs are theoretically analyzed in combination with experimental observations.

To analyze the peaks of monolayer and bilayer graphene RTTs, the numerical calculations described in Supplementary Section 4 were used, and the parameters were set as follow: γ = 10 meV; twist angle = 0°; d_{SiO_2} = 300 nm; ε_{SiO_2} = 3.9; d_{hBN} = 1.34 nm (for four-layer h-BN); ε_{hBN} = 3.58 (for four-layer h-BN; based on ref. [1]); T = 10 K. The calculation results are shown in Supplementary Fig. 15. The PVRs of the peak for the monolayer graphene and the near peak for the bilayer graphene were strongly affected by V_g, and the NDR at one V_d polarity was not obtained for approximately half the number of the V_g values. The PVRs of the far peaks for bilayer graphene were also affected by V_g but less significantly, and the NDR at two V_d polarities was obtained for all V_g values. This is because the monolayer graphene peak and bilayer graphene near peak both require additional V_g to achieve the resonant tunneling condition, whereas the bilayer graphene far peaks do not. The V_g-assisted resonant tunneling is based on the quantum capacitance effect. The quantum capacitance per unit area $C_{q,MG}$ for the monolayer graphene and $C_{q,BG}$ for the bilayer graphene are related to the Fermi level E_F:

$$C_{q,MG}\left(E_F\right) = e^2\left|\frac{dn_{MG}}{dE_F}\right| = \frac{2e^2|E_F|}{\pi\hbar^2 v_F^2}, \tag{28}$$

$$C_{q,BG}\left(E_F\right) = e^2\left|\frac{dn_{BG}}{dE_F}\right| = \begin{cases} \dfrac{2e^2\left(|E_F| + \gamma_1/2\right)}{\pi\hbar^2 v_F^2}, & |E_F| < \gamma_1 \\ \dfrac{4e^2|E_F|}{\pi\hbar^2 v_F^2}, & |E_F| \geq \gamma_1 \end{cases}. \tag{29}$$

$C_{q,BG}$ is always greater than $C_{q,MG}$, especially near the Dirac point, where $C_{q,MG}$ tends to 0 and $C_{q,BG}$ is not less than a value of $\left(e^2\gamma_1\right)/\left(\pi\hbar^2 v_F^2\right)$. Hence, the calculated bilayer graphene near peak had a lower voltage and PVR than the monolayer graphene peak with the same calculation parameters except for the number of graphene layers, as shown in Supplementary Fig. 15. The lower voltage for the bilayer graphene near peak agrees well with the experimental results shown in Fig. 1c,f and Supplementary Fig. 12a,b. Moreover, compared with the experimental results, the theoretical calculations overestimated the PVR of the bilayer graphene far peak, probably because of an additional Fowler–Nordheim tunneling component introduced by large bias, which was not considered in the calculations.

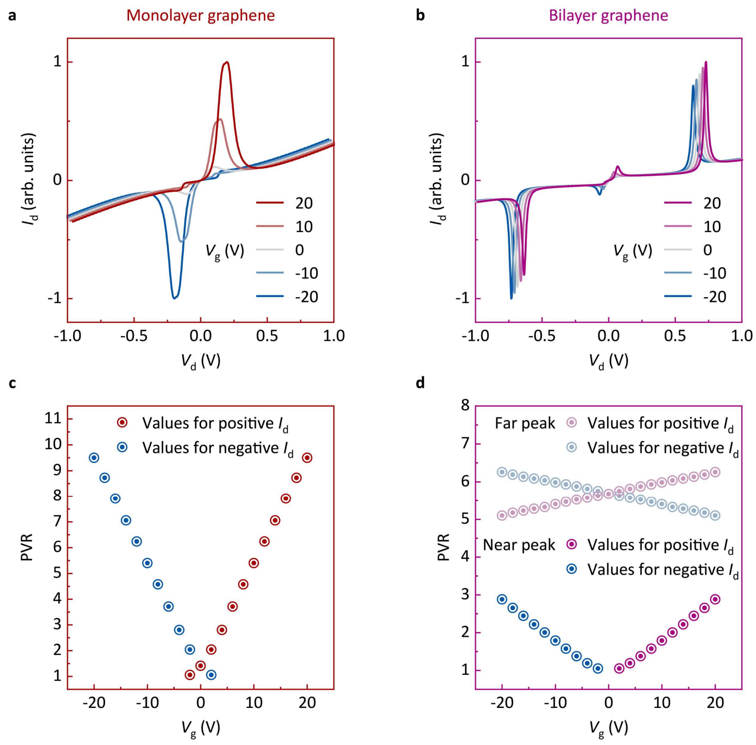

Supplementary Figure 15 | Calculation results for the peak comparison of the monolayer and bilayer graphene RTTs. a–**d**, Output characteristics (**a**,**b**) and PVRs (**c**,**d**) of monolayer (**a**,**c**) and bilayer (**b**,**d**) graphene RTTs.

To analyze the steps of monolayer and bilayer graphene RTTs, the output characteristics and the distributions of three energies, $E_{F,T}$, $E_{F,B}$, and $\Delta\varphi/2$, were calculated as shown in Supplementary Fig. 16. The calculation parameters were the same as those in Supplementary Fig. 15. For bilayer graphene RTT, in addition to the near and far steps, it was also predicted that two steps would be observed near $V_d = \pm0.6$ V, which we refer to as the middle steps. However, for 300-nm-thick SiO_2 as the gate dielectric, $V_g \geq 100$ V or ≤ -100 V is required to observe the middle steps, which is not easy experimentally owing to breakdown of the dielectric. The condition that the energies must satisfy for each peak and step is labeled in Supplementary Fig. 16a,b. The conditions for the step of the monolayer graphene RTT and the near and middle steps of the bilayer graphene RTT involve the Fermi energy. These steps are affected by the Fermi–Dirac distribution and are therefore temperature dependent. By contrast, the conditions of the far steps of the bilayer graphene RTT do not involve the Fermi energy. Therefore, these steps are independent of temperature. The contour maps of $|dI_d/dV_d|$ are shown in Supplementary Fig. 17, where the steps and their temperature dependence can be observed more clearly. The calculation parameters were the same as those in Supplementary Fig. 15, except for $T = 300$ K for Supplementary Fig. 17c,d. The discussion of these steps agrees well with the experimental results shown in Fig. 4e and Supplementary Fig. 13.

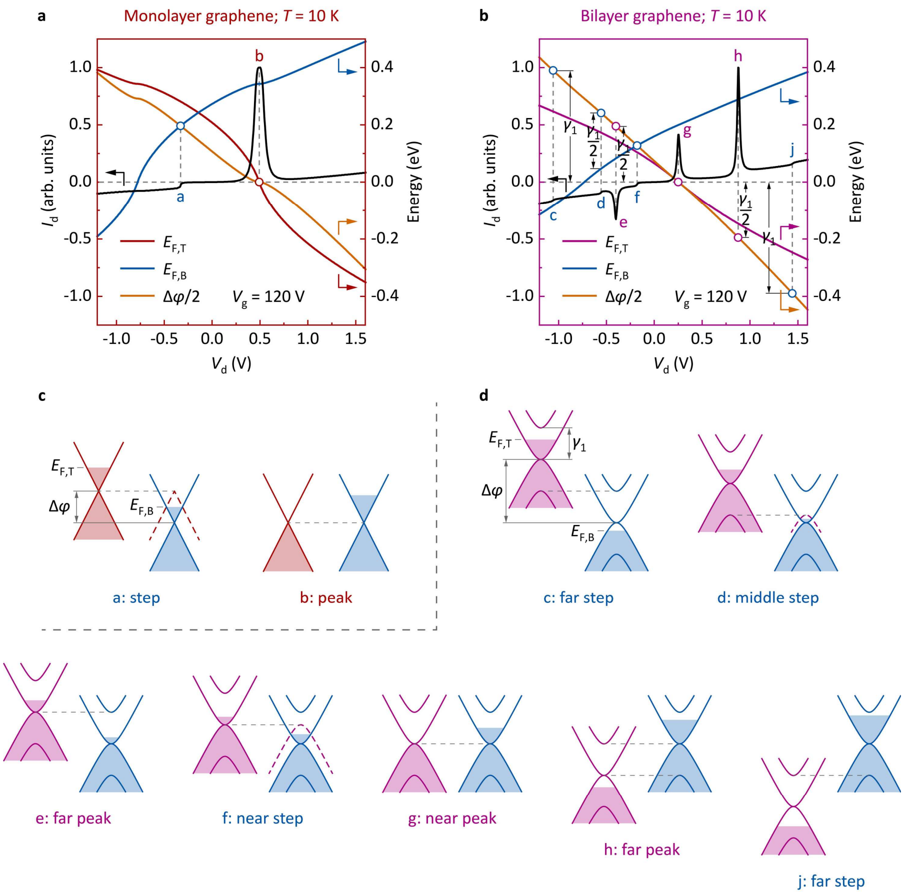

Supplementary Figure 16 | Conditions of the peaks and steps of the monolayer and bilayer graphene RTTs. a,**b**, Calculated output characteristics and energy distributions of monolayer (**a**) and bilayer (**b**) graphene RTTs under V_g = 120 V at 10 K. **c**,**d**, Band diagrams of monolayer (**c**) and bilayer (**d**) graphene RTTs for each peak and step.

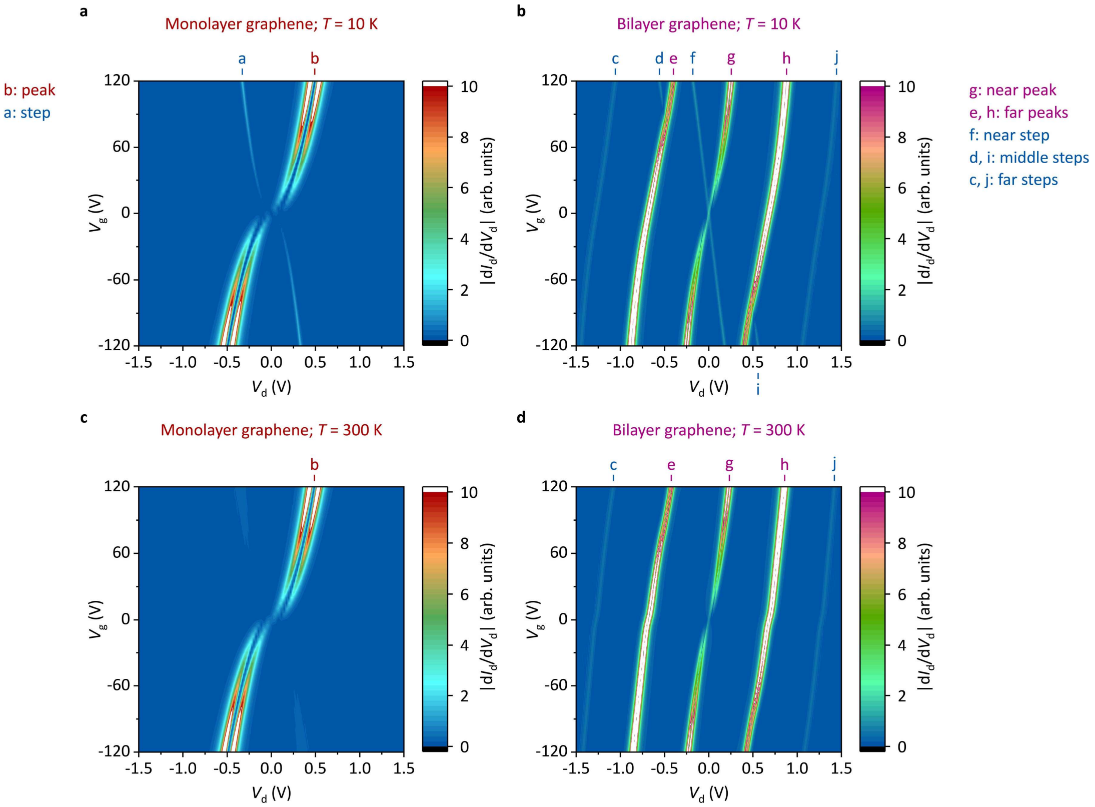

Supplementary Figure 17 | Calculation results for the step comparison of the monolayer and bilayer graphene RTTs. a–d, $|dI_d/dV_d|$ of monolayer (**a**,**c**) and bilayer (**b**,**d**) graphene RTTs at 10 K (**a**,**b**) and 300 K (**c**,**d**).

The peaks, steps, and intersections of the monolayer and bilayer graphene RTTs are summarized with the theory of the feature positions in Supplementary Table 1.

Supplementary Table 1 | Feature positions of monolayer and bilayer graphene RTTs

<table>
<tr><th colspan="6">Feature position: name | number</th><th>Condition</th><th>In this work or literature</th></tr>
<tr><td rowspan="2">Monolayer graphene RTT</td><td rowspan="2">2</td><td colspan="3">Peak</td><td>1</td><td>$\Delta\varphi = 0$</td><td>Fig. 1c, refs. [5–8]</td></tr>
<tr><td colspan="3">Step</td><td>1</td><td>$\Delta\varphi = 2E_{F,B}$</td><td>Fig. 4e, ref. [6] (with twist angle)</td></tr>
<tr><td rowspan="7">Bilayer graphene RTT</td><td rowspan="7">12</td><td rowspan="2">Peak</td><td rowspan="2">3</td><td>Near peak</td><td>1</td><td>$\Delta\varphi = 0$</td><td>Supplementary Fig. 12a, refs. [3,4,11,12]</td></tr>
<tr><td>Far peak</td><td>2</td><td>$\Delta\varphi = \pm\gamma_1$</td><td>Supplementary Fig. 12a, refs. [4,12]</td></tr>
<tr><td rowspan="3">Step</td><td rowspan="3">5</td><td>Near step</td><td>1</td><td>$\Delta\varphi = 2E_{F,B}$</td><td>Supplementary Fig. 13b, ref. [3]</td></tr>
<tr><td>Middle step</td><td>2</td><td>($\Delta\varphi = 2E_{F,B} + \gamma_1$ and $\Delta\varphi$, $E_{F,T}$, $E_{F,B} > 0$) or ($\Delta\varphi = 2E_{F,B} - \gamma_1$ and $\Delta\varphi$, $E_{F,T}$, $E_{F,B} < 0$)</td><td>Supplementary Figs. 16b and 17b (only in theory)</td></tr>
<tr><td>Far step</td><td>2</td><td>$\Delta\varphi = \pm 2\gamma_1$</td><td>Supplementary Fig. 13</td></tr>
<tr><td rowspan="2">Intersection</td><td rowspan="2">4</td><td>Near intersection</td><td>2</td><td>$\Delta\varphi \in (-\gamma_1, 0)$ or $\Delta\varphi \in (0, \gamma_1)$</td><td>Supplementary Fig. 12a,d</td></tr>
<tr><td>Far intersection</td><td>2</td><td>$\Delta\varphi < -\gamma_1$ or $\Delta\varphi > \gamma_1$</td><td>Supplementary Fig. 12a,d</td></tr>
</table>

Section 10 | Supplementary information of high-frequency testing

Supplementary Fig. 18 shows an optical micrograph and d.c. characteristics of the device shown in Fig. 5 after etching to a device area of S = 2.97 μm^2.

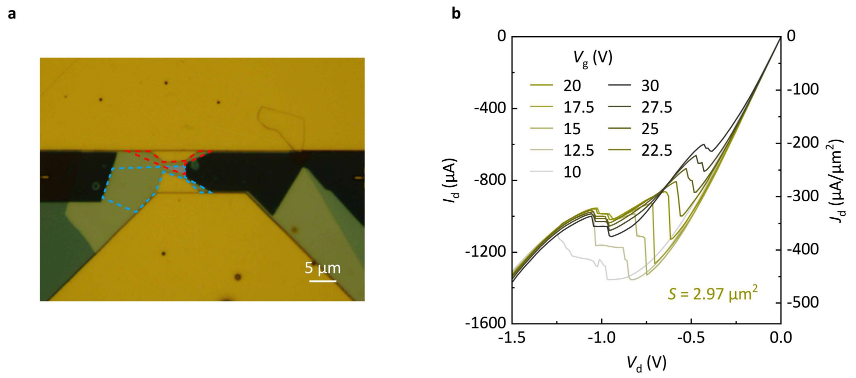

Supplementary Figure 18 | Optical micrograph and d.c. output characteristics of the bilayer h-BN barrier RTT for high-frequency testing. a, Optical micrograph. The red and blue dashed lines indicate the ranges of the top and bottom graphene flakes, respectively. **b**, Output characteristics in the d.c. test.

A monolayer graphene, trilayer h-BN barrier RTT was fabricated on a high-resistivity Si/SiO_2 substrate. Supplementary Fig. 19 shows the d.c. and high-frequency test results. The maximum operating frequency of 73 MHz was achieved in the high-frequency test at d.c. voltages of V_d = 0.37 V and V_g = 0 V. This value is much lower than the 11 GHz of the bilayer h-BN barrier device shown in Fig. 5.

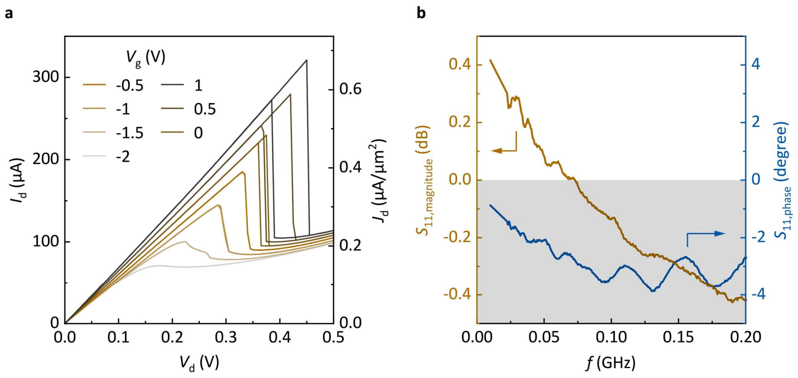

Supplementary Figure 19 | D.c. and high-frequency test results of a trilayer h-BN barrier RTT. a, Output characteristics in the d.c. test. **b**, Magnitude and phase of S_{11} as a function of the frequency f in the high-frequency test.

Section 11 | Area measurement of RTTs

The resolution of an optical microscope is $0.61\lambda/\mathrm{NA}$, where λ is the wavelength and NA is the numerical aperture. The resolution is generally hundreds of nanometers, leading to large errors when measuring RTTs with small sizes. Therefore, SEM was used to measure device areas below 10 μm^2. Supplementary Fig. 20 shows SEM images of two example devices.

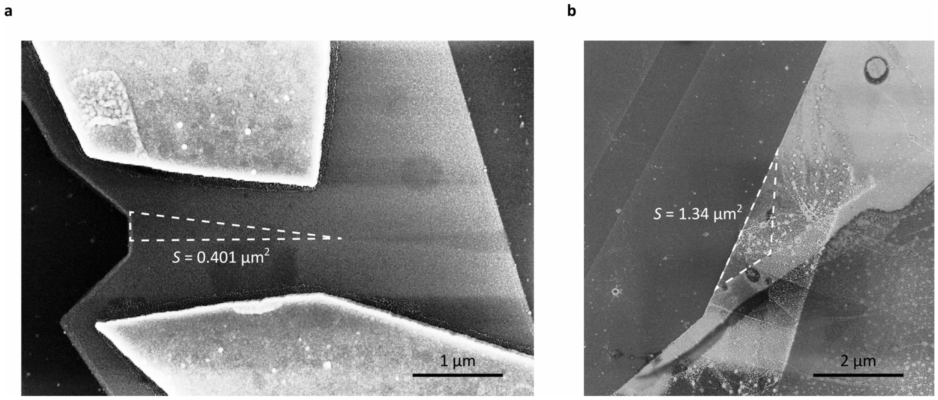

Supplementary Figure 20 | Examples of RTT area measurement using SEM. a,**b**, SEM images of the monolayer graphene, monolayer h-BN barrier device shown in Fig. 4a (**a**) and the monolayer graphene, bilayer h-BN barrier device shown in Fig. 2e (**b**).

Supplementary References

1. Laturia, A., Van de Put, M.L. & Vandenberghe, W.G. Dielectric properties of hexagonal boron nitride and transition metal dichalcogenides: from monolayer to bulk. *npj 2D Mater. Appl.* 2, 6 (2018).
2. Wang, D. et al. Thermally Induced Graphene Rotation on Hexagonal Boron Nitride. *Phys. Rev. Lett.* 116, 126101 (2016).
3. Burg, G. W. et al. Coherent Interlayer Tunneling and Negative Differential Resistance with High Current Density in Double Bilayer Graphene–WSe_2 Heterostructures. *Nano Lett.* 17, 3919–3925 (2017).
4. Kang, S. et al. Bilayer Graphene-Hexagonal Boron Nitride Heterostructure Negative Differential Resistance Interlayer Tunnel FET. *IEEE Electron Device Lett.* 36, 405–407 (2015).
5. Britnell, L. et al. Resonant tunnelling and negative differential conductance in graphene transistors. *Nat. Commun.* 4, 1794 (2013).
6. Mishchenko, A. et al. Twist-controlled resonant tunnelling in graphene/boron nitride/graphene heterostructures. *Nature Nanotechnol.* 9, 808–813 (2014).
7. Kuzmina, A. et al. Resonant Light Emission from Graphene/Hexagonal Boron Nitride/Graphene Tunnel Junctions. *Nano Lett.* 21, 8332–8339 (2021).
8. Zhang, Z. et al. Toward High-Peak-to-Valley-Ratio Graphene Resonant Tunneling Diodes. *Nano Lett.* 23, 8132–8139 (2023).
9. Martin, J. et al. Observation of electron–hole puddles in graphene using a scanning single-electron transistor. *Nat. Phys.* **4**, 144–148 (2008).
10. Kliros, G. S. A Phenomenological Model for the Quantum Capacitance of Monolayer and Bilayer Graphene Devices. *Romanian Journal of Information Science and Technology* **13**, 332–341 (2010).
11. Fallahazad, B. et al. Gate-Tunable Resonant Tunneling in Double Bilayer Graphene Heterostructures. *Nano Lett.* 15, 428–433 (2015).
12. Kim, K. et al. van der Waals Heterostructures with High Accuracy Rotational Alignment. *Nano Lett.* 16, 1989–1995 (2016).
